# Synthesis and characterization of iron clusters with an icosahedral [Fe@Fe_12_]^16+^ Core

**DOI:** 10.1093/nsr/nwad327

**Published:** 2023-12-22

**Authors:** Gan Xu, Yun-Shu Cui, Xue-Lian Jiang, Cong-Qiao Xu, Jun Li, Xu-Dong Chen

**Affiliations:** Jiangsu Collaborative Innovation Center of Biomedical Functional Materials and Jiangsu Key Laboratory of New Power Batteries, School of Chemistry and Materials Science, Nanjing Normal University, Nanjing 210023, China; Department of Chemistry and Guangdong Provincial Key Laboratory of Catalytic Chemistry, Southern University of Science and Technology, Shenzhen 518055, China; Department of Chemistry and Guangdong Provincial Key Laboratory of Catalytic Chemistry, Southern University of Science and Technology, Shenzhen 518055, China; Department of Chemistry and Guangdong Provincial Key Laboratory of Catalytic Chemistry, Southern University of Science and Technology, Shenzhen 518055, China; Department of Chemistry and Guangdong Provincial Key Laboratory of Catalytic Chemistry, Southern University of Science and Technology, Shenzhen 518055, China; Department of Chemistry and Engineering Research Center of Advanced Rare-Earth Materials of Ministry of Education, Tsinghua University, Beijing 100084, China; Jiangsu Collaborative Innovation Center of Biomedical Functional Materials and Jiangsu Key Laboratory of New Power Batteries, School of Chemistry and Materials Science, Nanjing Normal University, Nanjing 210023, China; State Key Laboratory of Coordination Chemistry, Nanjing University, Nanjing 210093, China

**Keywords:** Fe_13_ cluster, iron-sulfur cluster, low-valence iron, iron−iron bond, high spin complex

## Abstract

Iron-metal clusters are crucial in a variety of critical biological and material systems, including metalloenzymes, catalysts, and magnetic storage devices. However, a synthetic high-nuclear iron cluster has been absent due to the extreme difficulty in stabilizing species with direct iron−iron bonding. In this work, we have synthesized, crystallized, and characterized a (Tp*)_4_W_4_S_12_(Fe@Fe_12_) cluster (Tp* = tris(3,5-dimethyl-1-pyrazolyl)borate(1−)), which features a rare trideca-nuclear, icosahedral [Fe@Fe_12_] cluster core with direct multicenter iron−iron bonding between the interstitial iron (Fe_i_) and peripheral irons (Fe_p_), as well as Fe_p_···Fe_p_ ferromagnetic coupling. Quantum chemistry studies reveal that the stability of the cluster arises from the 18-electron shell-closing of the [Fe@Fe_12_]^16+^ core, assisted by its bonding interactions with the peripheral tridentate [(Tp*)WS_3_]^4−^ ligands which possess both S→Fe donation and spin-polarized Fe−W σ bonds. The ground-state electron spin is theoretically predicted to be S = 32/2 for the cluster. The existence of low oxidation-state (OS ∼ +1.23) iron in this compound may find interesting applications in magnetic storage, spintronics, redox chemistry, and cluster catalysis.

## Teaser

A low-valent iron cluster with icosahedral [Fe@Fe_12_]^16+^ core and multicenter iron-iron bonding has been synthetically achieved and computationally studied.

## INTRODUCTION

Clusters with precise composition and geometric structure represent the intermediate state between atoms/molecules and condensed-phase matter. The in-depth understanding of cluster assembly, structure, and properties can provide key information for bulk materials. Cluster science is therefore of fundamental interest for the development of innovative materials [1–6]. As one of the most important elements on earth, iron has played critical roles in natural metalloenzymes such as nitrogenase and hydrogenase [7–9], as well as in key industrial processes such as the Haber-Bosch and Fischer-Tropsch processes [10,11]. Iron-metal clusters have been extensively studied due to their promising magnetic properties with such potential applications as magnetic storage [12–16]. They provide intrinsic information on how iron atoms are assembled through iron−iron interactions, which provides a fundamental understanding of how physical properties such as magnetism evolve from iron atoms to bulk materials [12–16]. However, most experimental studies of iron-metal clusters focus on gas-phase species using techniques such as molecular beam deflection or low-temperature matrix isolation [13,14,17–19]. While it is challenging to provide precise atomic structure of iron clusters in such experiments, synthetic iron clusters are essential as the precise cluster composition and crystal structure provide rich information on iron−iron bonding and inter-cluster interactions.

However, the synthesis of iron clusters relies exclusively on ligand bridging. Usually, peripheral ligands help to link the iron atoms and stabilize the whole cluster [20–24], and internal ligands are the key to bridging the iron centers to form high-nuclear clusters [25–27]. Direct iron−iron bonding between the interstitial and peripheral iron atoms not assisted by ligand-bridging is a prerequisite for assembling a high-nuclear pure iron-metal cluster core. To date, unsupported iron−iron bonding has only been observed in very limited cases of dimeric iron complexes [28–30], whereas a high-nuclearity synthetic iron cluster with pure [Fe_n_] (*n* > 8) metal core is absent. The synthesis of high-nuclear iron-metal clusters has been challenging, hindering the understanding of the intrinsic nature of unsupported iron−iron bonding in iron materials. We report here the synthesis of an unprecedented icosahedral [Fe@Fe_12_] cluster compound featuring unsupported iron−iron bonding between the interstitial and peripheral iron atoms, in which the Fe oxidation state (OS) is +16/13 on average. This highly symmetric [Fe_13_] cluster, characterized by experimental techniques and quantum theoretical calculations, is the first crystallized iron cluster showing the precise atomic arrangement of the icosahedral [Fe_13_] core.

## RESULTS AND DISCUSSION

### Synthesis

**Figure 1.**
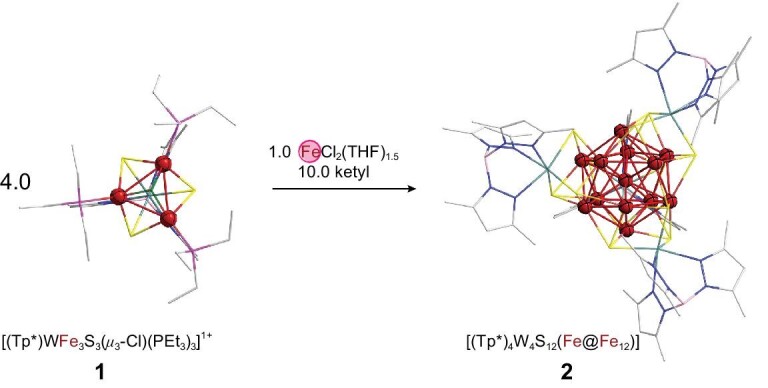
Synthesis of [(Tp*)_4_W_4_S_12_(Fe@Fe_12_)] **(2)**.

In our experiment, the discrete iron ions were first assembled into [Fe_3_] moieties using a template, which were further mounted around an interstitial iron atom under reduced conditions to form the [Fe@Fe_12_]-containing cluster. Three equivalents of iron(II) were readily assembled into the [(Tp*)WFe_3_S_3_(μ_3_-Cl)Cl_3_]^2−^ (Tp* = tris(3,5-dimethyl-1-pyrazolyl)borate(1−)) cluster containing a [Fe_3_] moiety [31], and then redox-free terminal ligand substitution with triethylphosphine ligands afforded [(Tp*)WFe_3_S_3_(*μ*_3_-Cl)(PEt_3_)_3_]^1+^ (**1**) as dark red crystals. Here the [(Tp*)WS_3_]^4−^ moiety retains the low-valence W(III) and Fe(II) in **1** as in the precursor. Accordingly, the addition of sodium benzophenone ketyl solution (10 equiv.) into the premixed tetrahydrofuran (THF) solution of **1** (4 equiv.) and FeCl_2_(THF)_1.5_ (1 equiv.) followed by pentane diffusion, resulted in the formation of diamond-shaped brown-black crystals. This crystalline product was characterized by single-crystal X-ray diffraction to be a high-nuclearity neutral cluster [(Tp*)_4_W_4_S_12_Fe_13_] (**2**, 41% average yield based on eight experiments, highest yield at 52%), which features four [(Tp*)WS_3_]^4−^ ligands around the [Fe_13_] cluster core. The synthetic process and structure of **2** is shown in Fig. 1. More experimental details are shown in Figs S1–S8 and Tables S1–S5 in the Supplementary Materials (SM).

The significant stability of the icosahedral [Fe@Fe_12_] assembly in **2** is intriguing. It appears that the reduction of the iron centers in the ferrous [WFe_3_]^9+^ core of **1** and FeCl_2_(THF)_1.5_ to lower oxidation states triggers the direct iron−iron interaction in **2**, leading to the aggregation of four [(Tp*WS_3_)Fe_3_] moieties around a central iron to form the icosahedral [Fe@Fe_12_] cluster core. According to the stoichiometric control of the synthetic design, the metal centers in starting materials **1** and FeCl_2_(THF)_1.5_ were reduced by 10 electrons from formal [W_4_Fe_13_]^38+^ (four [WFe_3_]^9+^ plus one Fe^2+^) to [W_4_Fe_13_]^28+^ during the reaction process. Indeed, both XPS (Fig. S2) and quantum-chemical theoretical studies (*vide infra*) of compound **2** suggest a +3 oxidation state of the W centers, thus rendering the [Fe@Fe_12_]^16+^ core an extremely low average oxidation state of OS = +16/13 (∼ +1.23) for these 13 Fe atoms.

### Structure and bonding

**Figure 2.**
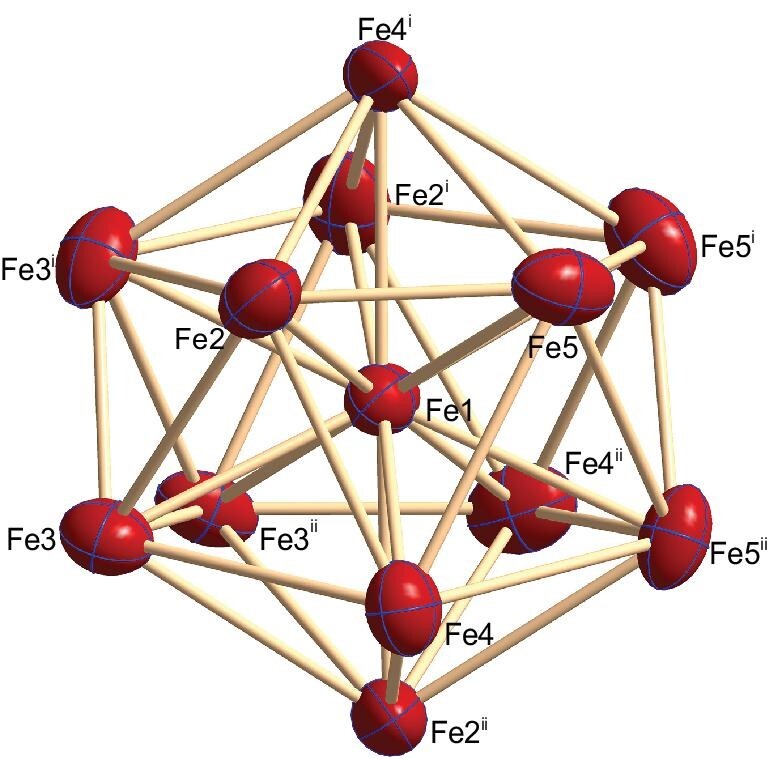
Structure of the [Fe@Fe_12_]^16+^ cluster. Thermal ellipsoids are set at the 65% probability level. Symmetry codes: i: -y, x-y, z; ii: y-x, -x, z. The Fe_p_−Fe_i_ bond lengths range from 2.483(1) to 2.530(1)  Å, with a mean value of 2.503 Å. The Fe_p_···Fe_p_ distances are between 2.377(1) and 2.793(1)  Å, averaged at 2.647 Å.

Compound **2** crystallized in the space group *R*$\bar{3}$, where the [Fe_13_]^16+^ core is surrounded by four [(Tp*)WS_3_]^4−^ species in a tetrahedral arrangement. Previous time-of-flight mass spectrometry studies and first-principles calculations showed that [Fe_13_] cluster corresponding to a magic number was likely stable, consistent with its more frequent occurrence in gas phase distribution [15,19,32–34]. Indeed, our experimental observations have shown that compound **2** with [Fe_13_]^16+^ core is a thermodynamically stable product frequently observed in various reactions. Due to steric restraints generated from the ligands and crystal packing, the molecular symmetry of **2** in the solid state is slightly lowered from ideal *T_d_* to its subgroup with a chiral *C_3_* symmetry, which has also slightly affected the *I_h_*-symmetry [Fe_13_]^16+^ core to become a distorted icosahedron. In this [Fe_13_]^16+^ cluster core, 12 peripheral iron atoms (Fe_p_) assemble around an interstitial iron atom (Fe_i_) to form an Fe-centered icosahedral [Fe@Fe_12_]^16+^ core. The determined structure of the [Fe@Fe_12_]^16+^ cluster core is shown in Fig. 2.

A typical structural feature of the [Fe@Fe_12_]^16+^ core is that the peripheral-interstitial Fe_p_−Fe_i_ bonds are unsupported, especially because they are completely ligand-free inside the Fe_12_ cage. Based on the quantum chemical calculations below, the Fe_p_−Fe_i_ bonds in the [Fe@Fe_12_]^16+^ core of compound **2** belong to delocalized multi-center Fe−Fe bonds. Meanwhile, the peripheral iron atoms are shown to interact with neighboring ones via ferromagnetic coupling. The Fe−S coordination and W−Fe bonding are found to be the major interactions between the surrounding [(Tp*)WS_3_]^4−^ species and the [Fe@Fe_12_]^16+^ core. Therefore, in compound **2**, the unsupported Fe_p_−Fe_i_ bonds and the Fe_p_···Fe_p_ ferromagnetic coupling account for the key factors that assemble the [Fe@Fe_12_]^16+^ aggregate, which was further stabilized by the surrounding [(Tp*)WS_3_]^4−^ species through Fe−S coordination and W−Fe bonding. In contrast, for the synthetic iron clusters with nuclearity <8, their cluster aggregation relies exclusively on Fe−Fe bonds bridged by external ligands and their stabilization requires extreme conditions such as −80°C in some cases [20–24].

Low-valence iron is potentially significant in crucial catalytic systems such as carbon fixation involving carbon monoxide dehydrogenase and nitrogen fixation utilizing nitrogenase [35,36]. From a synthetic point of view, such low-valence species have usually been stabilized by carefully designed low-coordinate phosphorus, nitrogen, and sulfur ligands [37–40]. The presence of low-valence iron in iron clusters is rather scarce [20,22,23]. The current case of compound **2** represents a unique example of a high nuclearity (>8) iron cluster containing multiple low-valence iron centers, which may correlate with versatile biomimetic and industrial processes.

### Mössbauer studies

**Figure 3.**
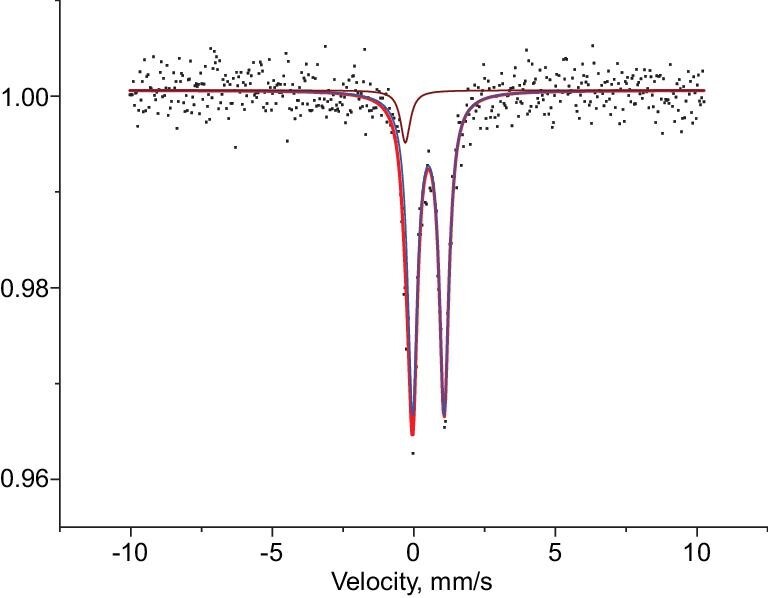
Zero field Mössbauer spectrum of **2** obtained at 77 K. Simulation parameters: *δ*/|*ΔE_Q_*| (mm/s): blue fit (92.3%): 0.49(1)/1.16(1); brown fit (7.7%), 0.04(2)/0(2).

The zero-field ^57^Fe Mössbauer spectrum of compound **2** at 77 K is shown in Fig. 3, which has a slightly asymmetric doublet. Structurally, all 12 peripheral iron atoms of the icosahedron adopt the same coordination environment while the interstitial Fe atom interacts only with the 12 peripheral Fe atoms and has a completely different environment. Therefore, a two-component fitting was applied to the Mössbauer spectrum. The major component (blue fit, 92.3%) exhibits an isomer shift of 0.49 mm/s and the corresponding quadrupole splitting is 1.16 mm/s, which is assigned to the peripheral iron atoms. The other component (brown fit, 7.7%) has an isomer shift of 0.04 mm/s and no obvious quadrupole splitting belonging to the interstitial iron is observed.

### Quantum chemistry studies

Quantum chemistry studies were performed to probe the geometric and electronic structures of the [Fe@Fe_12_]^16+^ core and compound **2** (the computational details are given in the SM). A simplified model **2-H** (see Fig. S9) with *T* symmetry was used as the model cluster, which is formed by replacing all methyl groups of **2** by hydrogen atoms to save computational time. The optimized structures of **2** and the simplified scheme **2-H** show almost negligible differences in geometry, both of which agree well with the experimental crystal structure, as illustrated in Table S6. A variety of spin-multiplicity states have been evaluated (see Fig. S10) and the ground state is computationally predicted to have a spin quantum number of S = 32/2 for **2**. While the particular spin multiplicity of the iron systems might change with different computational methods, the high spin feature hereby is remarkable.

**Figure 4.**
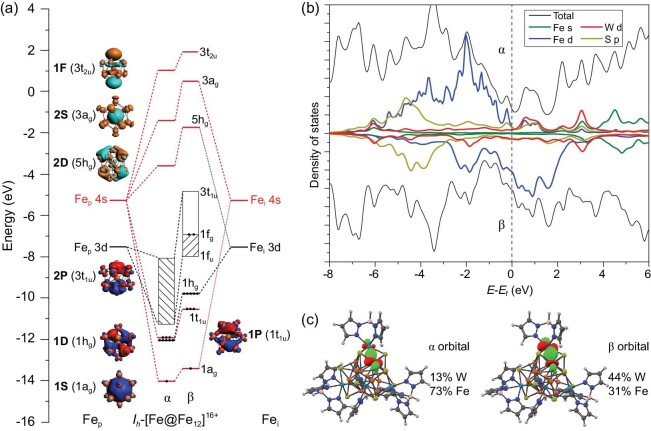
Electronic structure. (a) Schematic Kohn-Sham MO diagram of *I*_h_−[Fe@Fe_12_]^16+^ and contours of spherical jellium superatom orbitals. (b) Density of states (DOS) for **2-H**. (c) Contours of α and β Pipek-Mezey LMOs of the Fe−W σ bond at the PBE level [50].

As mentioned above, the [Fe@Fe_12_]^16+^ core of **2** has a pseudo-icosahedral structure, and the discussion will be based on the symmetrized *I_h_* model for brevity. We first studied the interaction between Fe_i_ and 12 Fe_p_ by the fragment molecular orbital (FMO) approach. As shown in Fig. 4a, the twelve 4s orbitals of Fe_p_ span *a*_g_(S), *t*_1u_(P) bonding group orbitals (GOs) and *h*_g_(D), *t*_2u_(F) antibonding GOs. The interaction between GOs of Fe_p_ and atomic orbitals (AOs) of Fe_i_ can be classified into superatomic S-type, P-type and D-type. Such interaction gives rise to a 1S^2^1P^6^1D^10^ configuration of spherical jellium orbitals for [Fe@Fe_12_]^16+^ by involving the 4s-based bonding GOs (*a*_g_, *t*_1u_) of Fe_p_, which results in occupied 1*a*_g_, 1*t*_1u_ and 1*h*_g_ orbitals that fulfills the ‘18-electron rule’. This 18-electron configuration provides considerable stability, as in the case of endohedral icosahedral M@Au_12_ (M = Mo, W) clusters [41,42]. The spin population of Fe_i_ is roughly 3, suggesting a 3d^7^ electronic configuration with an oxidation state of +1. This Fe(I) assignment is further supported by the calculated spin-polarized electron configuration of 3d^7.81(4.95α+2.86β)^ (Table S7) for Fe_i_ by natural population analysis (NPA) of [Fe@Fe_12_]^16+^, where the lower occupation number of β−spin 3d orbitals (*h*_g_ symmetry) than the α−spin can be attributed to their significant interaction with the 1*h*_g_ orbitals derived from Fe_p_−4s AOs.

The bonding scheme (Fig. 4) can well explain why the low-valence iron cluster can be stable through Fe−Fe direct bonding, as in the case of M^I^_8_ (M = Mn, Zn) clusters with cubic aromaticity [43,44]. The [(Tp*)_4_W_4_S_12_(Fe@Fe_12_)] cluster can be considered as being composed of four [(Tp*)W^III^S_3_(Fe^II^_3_)]^2+^ precursor fragments and a central Fe^2+^ ion to form the [(Tp*)_4_W^III^_4_S_12_(Fe^II^@Fe^II^_12_)]^10+^ cluster with an icosahedral (Fe@Fe_12_)^26+^ cluster core. Upon formation of this tridecanuclear iron cluster, the Fe 4s-orbitals will form bonding orbitals of *a*_g_(S)+*t*_1u_(P) GOs, while the five 3d-orbitals of the central Fe_i_ will be stabilized via orbital interaction with Fe 4s-based *h*_g_ GOs. The stabilization of these orbitals will cause the cluster to be reduced by 10 electrons via the formation of direct Fe−Fe bonds, which lead to the (Fe@Fe_12_)^16+^ cluster core with low OS = 16/13 (≈1.23).

On the other hand, besides the 18 electrons of the 1S^2^1P^6^1D^10^ superatomic configuration and the +16 charge for [Fe@Fe_12_]^16+^, 70 (N*_e_* = 8 × 13 − 18 − 16) electrons are left for the 3d ‘band’ composed of 60 Fe_p_ 3d-based orbitals. Notably, the 3d orbitals are rather radially contracted (Fig. S12) due to quantum primogenic effects and can hardly form covalent bonds with each other at a distance of ∼2.5 Å because of insufficient orbital overlapping. Instead, the Fe_p_−Fe_p_ interaction involves ferromagnetic coupling, leading to an energetically low-lying α-3d band stabilized by exchange interactions. The occupation pattern with 60 α electrons and 10 β electrons located at 60 3d-based Fe_p_ orbitals is favored for the pure [Fe@Fe_12_]^16+^ core, where the strong antibonding 3*t*_1u_(2P) orbitals possess the highest energy, as depicted in Fig. 4a. However, upon coordination by [(Tp*)WS_3_]^4−^ ligands, the 2P superatom orbitals of [Fe@Fe_12_]^16+^ in spin α will be strongly destabilized by the P-type ligand group orbitals (LGOs) to yield three vacant antibonding orbitals with high energies, thus promoting spin flip of three α electrons on the 2P orbitals of [Fe@Fe_12_]^16+^ to β set, resulting in the cluster electronic state of [Fe@Fe_12_]^16+^ to be S = 44/2 (Fig. S13).

Consequently, the interaction between the [Fe@Fe_12_]^16+^ core and the ligands [(Tp*)WS_3_]^4−^ was investigated by analyzing the density of states (DOS), as displayed in Fig. 4b. By comparing the DOS before and after ligation (Figs S14, S15), it can be found that the 3d band of Fe was broadened due to the interaction with the ligands and the increased distributions of β 3d states at the Fermi level, indicating the expected electron transfer from ligands. Additionally, principal interacting spin orbital (PISO) analysis [45] was carried out to extract the one-to-one interacting scenario between the [Fe@Fe_12_]^16+^ core and ligands. As illustrated in Table S8 and Figs S16 and S17, we identified two Fe−ligand interactions: the spin-polarized Fe−W interactions and the Fe−S interactions predominated by S-, P-, D-, F-type S→Fe donation. Furthermore, the bond distance of Fe−W (2.70 Å) is slightly longer than the sum of Pyykkö’s covalent radii for Fe and W atoms (2.53 Å) [46] and the calculated bond order is more than 0.50 (Table S1), indicating the existence of non-negligible Fe−W bonding. The DOS of W mainly distributed at ∼0.8 eV above the Fermi level for α states and ∼2.0 eV below the Fermi level for β states. Besides, the Fe−W σ bonds for spin α are Fe-dominated while remarkable increases of W contributions are found for spin β in the localized molecular orbitals (LMOs) (see Fig. 4c and Fig. S8), further demonstrating the spin-polarized character of the Fe−W bonds, which can also be captured in the spin density population in Fig. S19. Therefore, besides the intrinsic stability of the [Fe@Fe_12_]^16+^ core, the enhanced stability of **2** can be attributed to interactions from the ligands. First, electron donation from sulfur-based ligands stabilizes the Lewis-acidic sites of the Fe_13_ cluster core. Meanwhile, the paramagnetic W(III) centers are ‘magnetized’ by the high-spin Fe_13_ core, providing magnetic attachment between Fe and W, as shown in the spin-polarized Fe−W σ bonds. Both types of attractions enable the ligand-induced stabilization of the Fe_13_ core in **2**.

To gain a quantitative and chemically intuitive understanding of the spin polarization of the Fe−W bonds, we also performed a spin natural orbital (SNO) analysis [47], where a larger absolute SNO eigenvalue |λ| corresponds to the bigger density difference between α and β densities and therefore stronger spin polarization. As shown in Fig. S20, 12 dominant SNO pairs with |λ| of 0.260–0.432 are assigned to 12 Fe−W bonds. Meanwhile, the exchange interaction between the α electron of the Fe−W bond and excess α electrons accumulated at the [Fe@Fe_12_]^16+^ core will stabilize the structure, which facilitates α spin density around Fe while inducing β spin density aggregation at the W atom. Thus, one can conclude that each W provides three β electrons and appears as W(III), while three adjacent Fe atoms offer three α electrons to form the spin-polarized Fe−W σ bonds, thus quenching 12 single electrons and leading to a ground state with S = 32/2 for **2-H**.

## CONCLUSIONS

In summary, an iron cluster with tridecanuclear [Fe@Fe_12_]^16+^ core featuring unsupported Fe_p_−Fe_i_ bonding has been synthesized. The [Fe@Fe_12_]^16+^ cluster core with precise atomic structure is the first synthetic icosahedral [Fe_13_] cluster. This unique [Fe@Fe_12_]^16+^ cluster with low-valence iron centers is assembled by the multi-center Fe_p_−Fe_i_ bonds and Fe_p_···Fe_p_ ferromagnetic coupling, and is further stabilized by the peripheral [(Tp*)WS_3_]^4−^ species through Fe−S coordination and W−Fe bonding. Theoretical studies indicate that the 18-electron structure of the icosahedral superatomic [Fe@Fe_12_]^16+^ core is responsible for the special intrinsic stability of the structure. The Fe−S interaction reveals S→Fe donation that polarized toward sulfur and the Fe−W interaction can be localized into 12 electron-sharing σ bonds with strong spin polarization. We also demonstrate that three β electrons of the W atom are involved in one Fe−W bond, leading to the oxidation state W(III) and the number of unpaired electrons for compound **2** to be 32. The special stability of this [Fe@Fe_12_] cluster bears resemblance in superatom characteristics to the recently found Rh@Rh_12_@Rh_6_ [48] and Co@Co_12_O_8_ clusters with cubic aromaticity [4]. The synthesis and crystallization of this Fe_13_ cluster underscores a feasible synthetic methodology toward reactive late transition-metal clusters with higher nuclearity.

## NOTE ADDED IN THE PROOF

We noticed a latest paper on the same structure published by Scott *et al.* in *Angew. Chem. Int. Ed.* [49] after the manuscript had been submitted. Our synthesis approach differs significantly from theirs in both the starting materials and the strategy of cluster assembly, indicating that an Fe_13_ cluster can be synthesized through different approaches.

## METHODS

Details of methods are included in the Supplementary data.

## DATA AVAILABILITY

Crystallographic data can be obtained free of charge from the Cambridge Crystallographic Data Centre (www.ccdc.cam.ac.uk/data_request/cif) with CCDC numbers 2106794(2) and 2106795(1).

## Supplementary Material

nwad327_Supplemental_File
