# Supplementary material for: Synthesis and characterization of iron clusters with an icosahedral [Fe@Fe12]16+ Core
Source: Natl Sci Rev. 2023 Dec 22;11(4):nwad327. doi: 10.1093/nsr/nwad327 (PMC10939364; doi:10.1093/nsr/nwad327)
Supplement: nwad327_Supplemental_File [file nwad327_supplemental_file.docx]

Supplementary Materials for

**Synthesis and Characterization of Iron Clusters with an Icosahedral [Fe@Fe_12_]^16+^ Core**

Gan Xu,^1†^ Yun-Shu Cui,^2†^ Xue-Lian Jiang,^2^ Cong-Qiao Xu,*^2^*

Jun Li, *^2,3^** Xu-Dong Chen*^1,4^**

Correspondence to: [junli@tsinghua.edu.cn](mailto:junli@tsinghua.edu.cn); [xdchen@njnu.edu.cn](mailto:xdchen@njnu.edu.cn)

**This PDF file includes:**

Materials and Methods

Supplementary Text

Figs. S1 to S20

Tables S1 to S8

References

General methods

All manipulations were performed under a dry nitrogen/ argon atmosphere in the glove box (MBRAUN or Vigor) or on a standard Schlenk line. Ether, hexane, pentane, and toluene were refluxed over sodium metal and benzophenone until dry and distilled under N_2_ atmosphere. THF(Tetrahydrofuran) was refluxed over potassium metal and benzophenone until dry and distilled under N_2_ atmosphere. Acetonitrile was refluxed over CaH_2_ for 24 hours and distilled under N_2_ atmosphere. N,N-Dimethylformamide (DMF) was dried by CaH_2_ at 60 ^o^C for 24 hours under N_2_ atmosphere and distilled under reduced pressure at 60 ^o^C. All the solvents were degassed prior to use and stocked over 3Å molecular sieves in the glove box. (Et_4_N)_2_[(Tp*)WFe_3_S_3_(μ_3_-Cl)Cl_3_] and FeCl_2_(THF)_1.5_ were synthesized by published methods [1, 2]. All other chemicals were commercially available from J&K, Alfa Aesar, Sigma Aldrich, and Aladdin.

Physical Measurements

^1^H NMR data were obtained with Bruker Avanced 400 MHz spectrometer. All ^57^Fe Mossbauer spectroscopies were collected with a constant-acceleration spectrometer at 77 K (WSS-10 Mössbauer spectrometer). Isomer shifts were referenced to α-Fe at room temperature. Mössbauer spectra were fitted using the Moss Winn 4.0 program at the Dalian Institute of Chemical Physics (CAS). The ESI mass spectrometry was conducted on Thermo Scientific Q Exactive at the State Key Laboratory of Coordination Chemistry, Nanjing University. Elemental analyses were performed by the Analytical Laboratory of the Shanghai Institute of Organic Chemistry (CAS). XPS was performed by Thermo Scientific K-Alpha+.

Synthesis

**1**•(BPh_4_) **----** [(Tp*)WFe_3_S_3_(μ_3_-Cl)(PEt_3_)_3_] (BPh_4_)

(Et_4_N)_2_[(Tp)*WFe_3_S_3_(μ_3_-Cl)Cl_3_] (56.2 mg, 0.05 mmol) was suspended in 5.0 ml THF. Then, PEt_3_ stock solution (150 μL, 1M THF solution) was transferred into the suspension, followed by the addition of Na(BPh_4_) (51.3 mg, 0.15 mmol). The reaction mixture was stirred overnight, filtrated through celite, and diffused with diethyl ether to afford dark red block-like crystals (60.0 mg, 83%).

^1^H NMR (CD_3_CN, 400 MHz, δ, ppm): 45.55 (br, 6H, CH_2_), 0.35 (br, 9H, CH_3_), -14.04 (3H, CH_3_), 14.45 (br, H, CH)

Anal. Calcd for **1**•(BPh_4_) (C_57_H_87_B_2_ClFe_3_N_6_P_3_S_3_W): C, 47.09; H, 6.03; N, 5.78. Found: C, 46.91; H, 6.05; N, 5.85.

**2 ----**[(Tp*)_4_W_4_S_12_Fe_13_]

**1**•(BPh_4_) (72.7 mg, 0.05 mmol) was dissolved in 4 mL THF, to which was added FeCl_2_(THF)_1.5_ (3.1 mg, 0.0125 mmol) in 1 mL THF. After 30 minutes of stirring, sodium benzophenone ketyl solution (1.25 mL, 0.1M) was transferred into the solution. After another 30 minutes of stirring, the reaction mixture was filtrated through Celite, and the filtrate was diffused by pentane to give **2**•(THF)_4.5_ (17.6 mg, 41%).

No NMR spectrum is available due to the limited solubility of **2** in deuterated THF and CD_3_CN.

Anal. Calcd for **2•**THF (C_64_H_96_B_4_Fe_13_N_24_OS_12_W_4_): C, 24.74; N, 10.82; H, 3.11; Found: C, 24.49; N, 10.33; H, 3.17.

**Note for the reductant**: We had conducted extensive experiments using various potent reductants. The utilization of heterogeneous Sodium (Na) and Potassium (K) resulted in a complex array of products, complicating the purification process. This is likely due to an over-reduction scenario. With Potassium Graphite (KC_8_), we did produce the desired product, but the yield was unfortunately suboptimal. While we also tried Potassium Anthracene as a substitute for the ketyl solution, this approach also led to lower yields of the target products. Despite our rigorous attempts with different ratios and types of potent reductants, we were unable to isolate any iron clusters at different metal oxidation states using these reductants.

Mass spectrometry


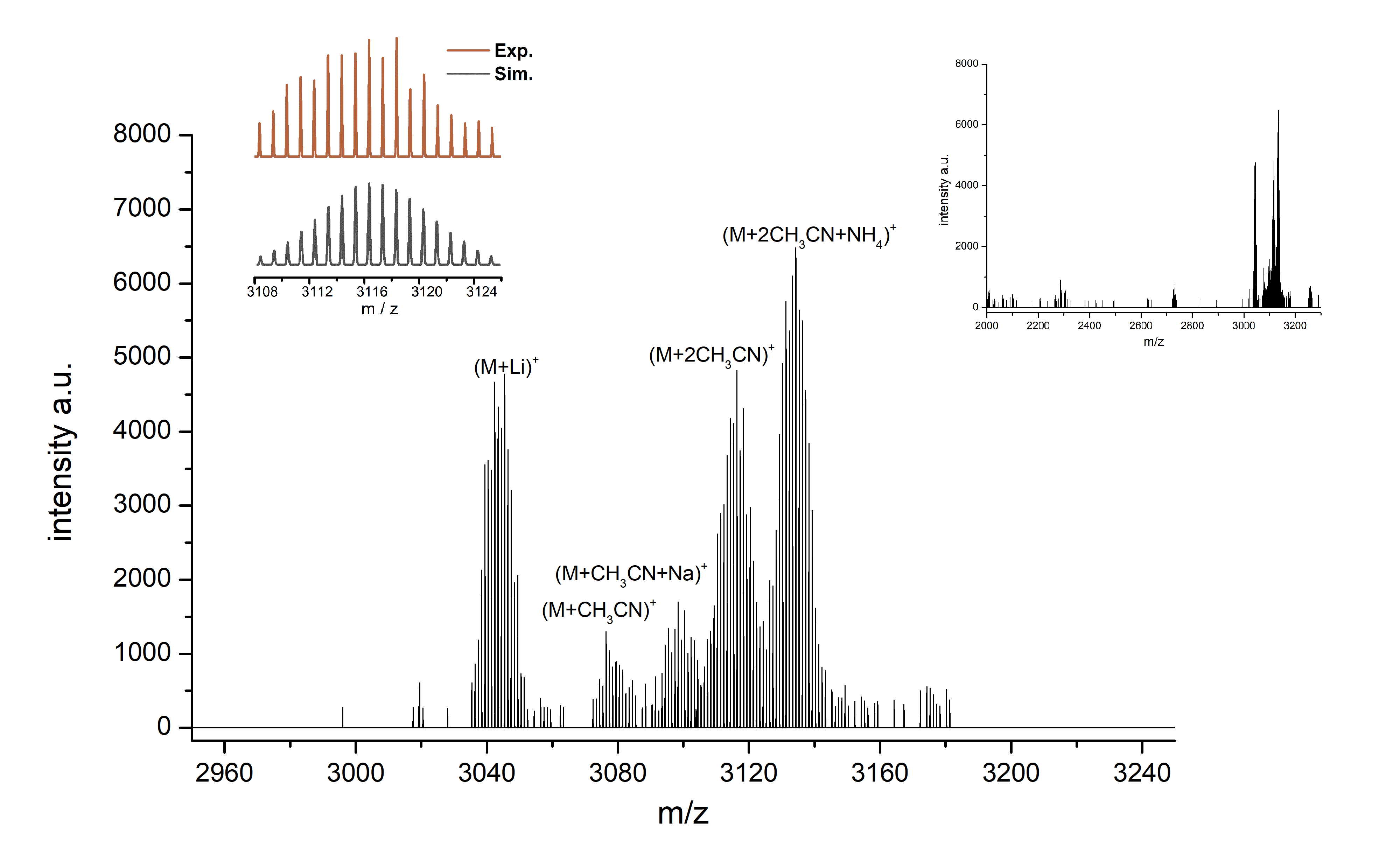
**Fig. S1.** High-resolution ESI-MS spectrum of the as-synthesized **2**; left inset: experiment and theoretical isotopic distribution spectra; right inset: full-scale ESI-MS spectrum.

XPS spectrometry


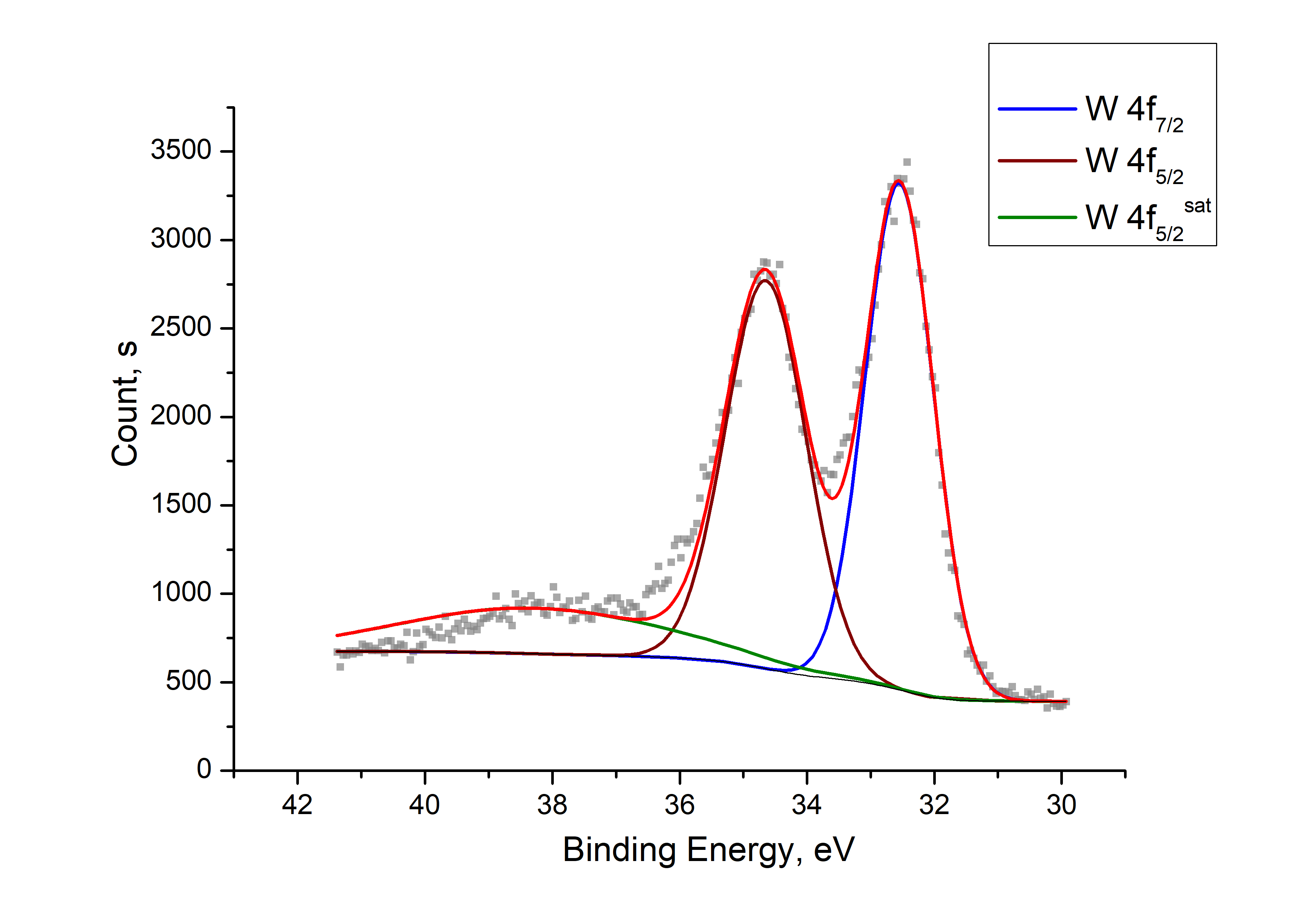


**Fig. S2**. The XPS spectra of cluster **2** at the W4f_7/2,5/2_ level.

**Table S1.** W4f_7/2,5/2_ binding energies from the cluster **2**

| Sample | W4f_7/2_ | W4f_5/2_ | W4f_5/2_^sat^ |
| --- | --- | --- | --- |
| **2** | 32.55 eV | 34.64 eV | 38.28eV |

UV−vis Absorption Spectrum


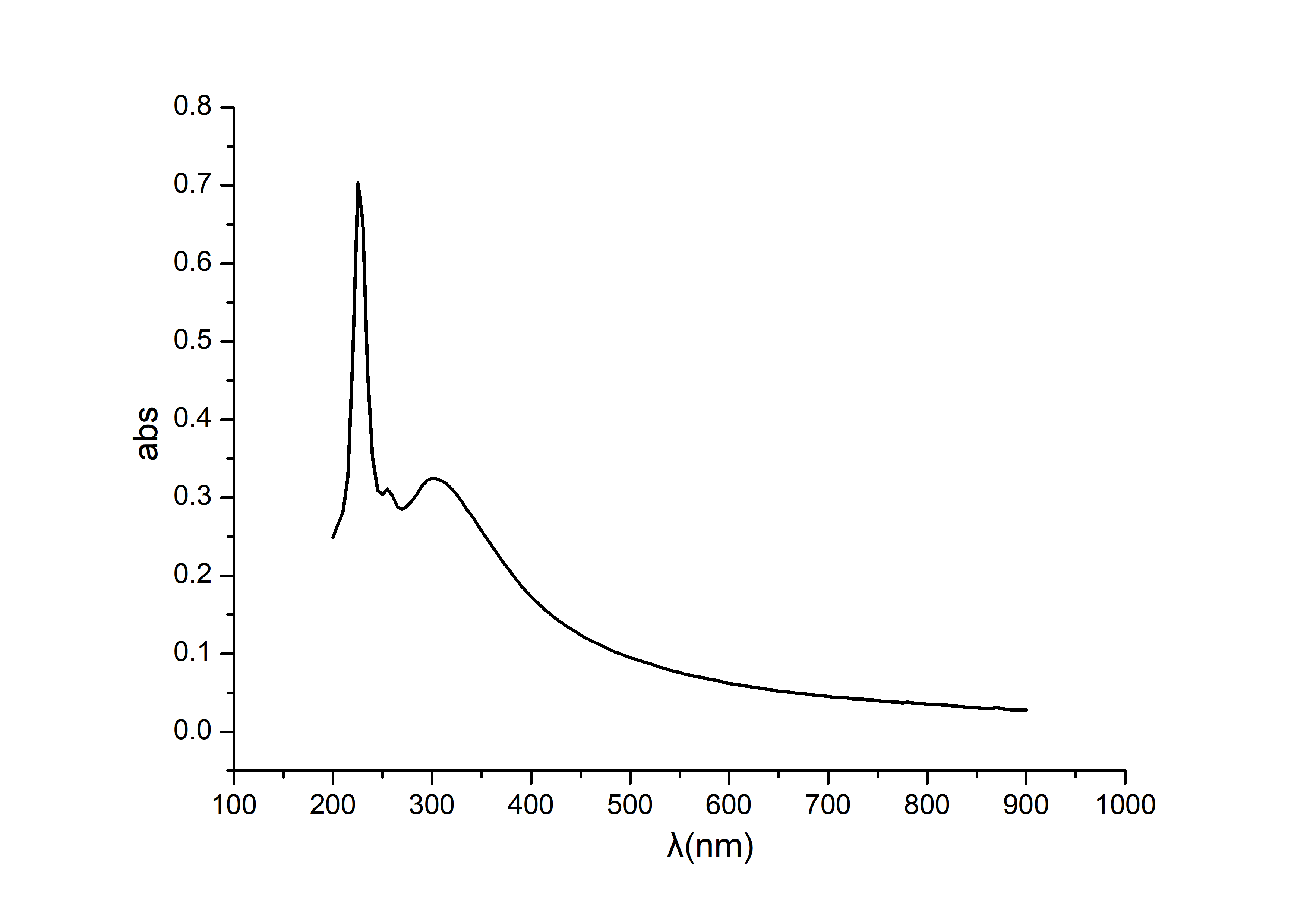


**Fig. S3**. Solution UV−vis absorption spectrum of **2** in THF.

Comparison of mean Fe-Fe distance in iron clusters

**Table S2.** Comparison of mean distance of Fe-Fe in iron clusters

| Sample | Fe-Fe distance(Å) | reference |
| --- | --- | --- |
| **2** | 2.503(1)^a^, 2.648(1)^b^ | This work. |
| [(18-C-6)K(thf)_2_][(^tbs^L)Fe_3_] | 2.446(1) | [3] |
| [(^tbs^L)Fe_3_] | 2.577(1) | [4] |
| (HL)_2_Fe_6_ | 2.597(1) | [5] |
| [(HL)_2_Fe_6_][BarF_24_] | 2.608(11) | [6] |
| [Bu_4_N][(HL)_2_Fe_6_] | 2.599(11) | [6] |
| [Bu_4_N]_2_[(HL)_2_Fe_6_] | 2.573(6) | [6] |
| [MgCl(THF)_5_][Fe_8_Me_12_] | 2.433(1) | [7] |
| [MgX(THF)_5_]_2_[Fe_8_Et_12_] | 2.418(1) | [8] |
| Fe_3_(COT)_3_ | 2.824(1) | [9] |
| [Na_12_Fe_6_(tris-cyclosalophen)_2_(THF)_14_] | 2.421(1)^c^, 2.703(1)^d^ | [10] |

^a^ Fe_p_-Fe_i_ distance, ^b^ Fe_p_-Fe_p_ distance, ^c^ N bridging Fe-Fe distance, ^d^ O bridging Fe-Fe distance.

Diagrams showing the crystal structure of cluster [(Tp*)_4_W_4_S_12_Fe_13_] (2)


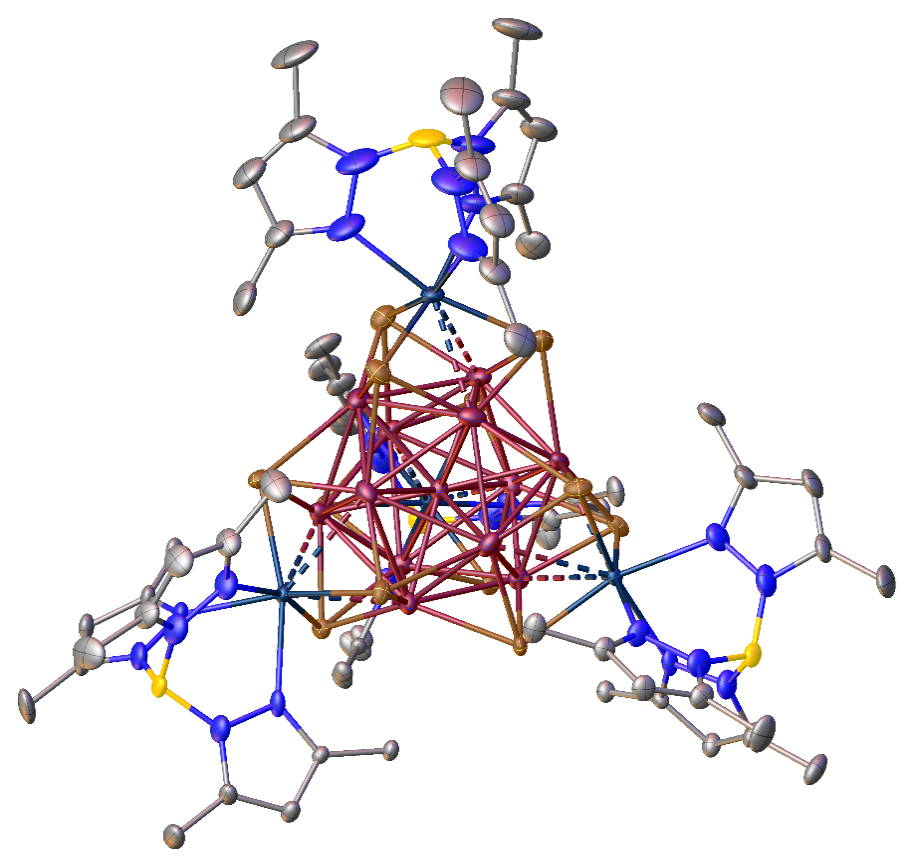


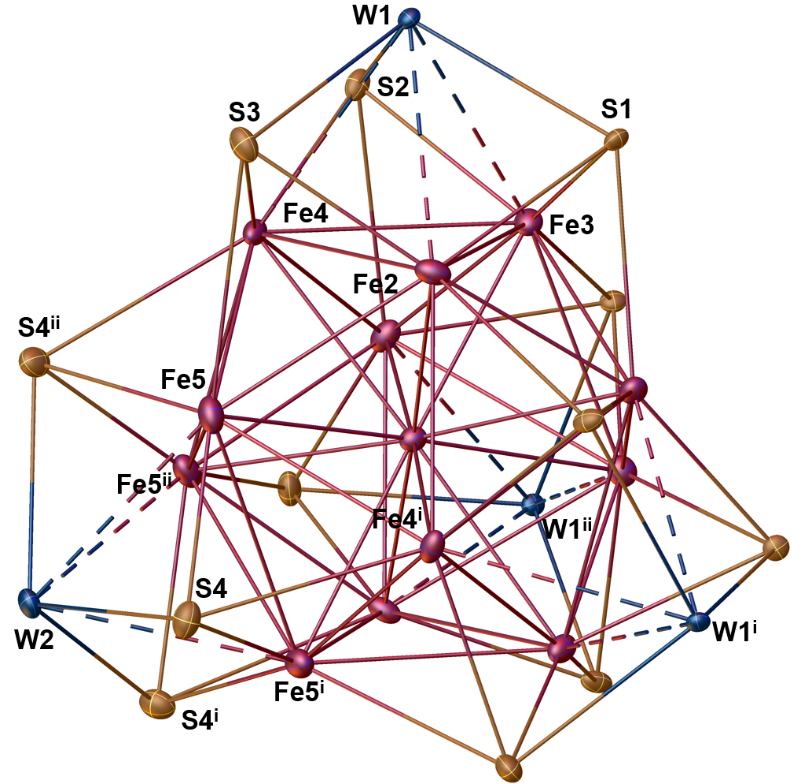


**Fig. S4.** Crystal structure of cluster **2** (top) and the inner core of [W_4_S_12_Fe_13_] (bottom)

As shown in **Fig. S5**, a 3-fold axis passes through B2-W2-Fe1 and the centroids of two opposite [Fe_3_] triangles (Fe3-Fe3^i^-Fe3^ii^ and Fe5-Fe5^i^-Fe5^ii^, symmetry code: i: -y, x-y, z; ii: y-x, -x, z). The Fe_p_-Fe_i_ bond lengths range from 2.483(1) to 2.530(1) Å, with a mean value of 2.503 Å. The Fe_p_···Fe_p_ distances are between 2.377(1) and 2.793(1) Å, averaged at 2.647 Å. From a coordination point of view, the twenty [Fe_3_] triangles of the distorted icosahedron can be classified into three types. Four of the triangles (Type **I**) are each covered by one [(Tp*)WS_3_] moiety to form a tetrahedral [WFe_3_] unit with W-Fe distances between 2.692(3) Å and 2.734(3) Å (Fig. S6). Each sulfur atom of the [(Tp*)WS_3_] fragment binds to a [Fe_3_] triangle (Type **II**) neighboring the Type **I** [Fe_3_] triangle through Fe-S coordination, generating a distorted tetrahedral [SFe_3_] unit (Fig. S7). Twelve out of the twenty [Fe_3_] triangles belong to Type **II**. The other four [Fe_3_] triangles (Type **III**) have no direct cover on top of them and exhibit an open environment (Fig. S8), which are, however, quite inert toward N_2_ and CH_4_ according to theoretical calculation.


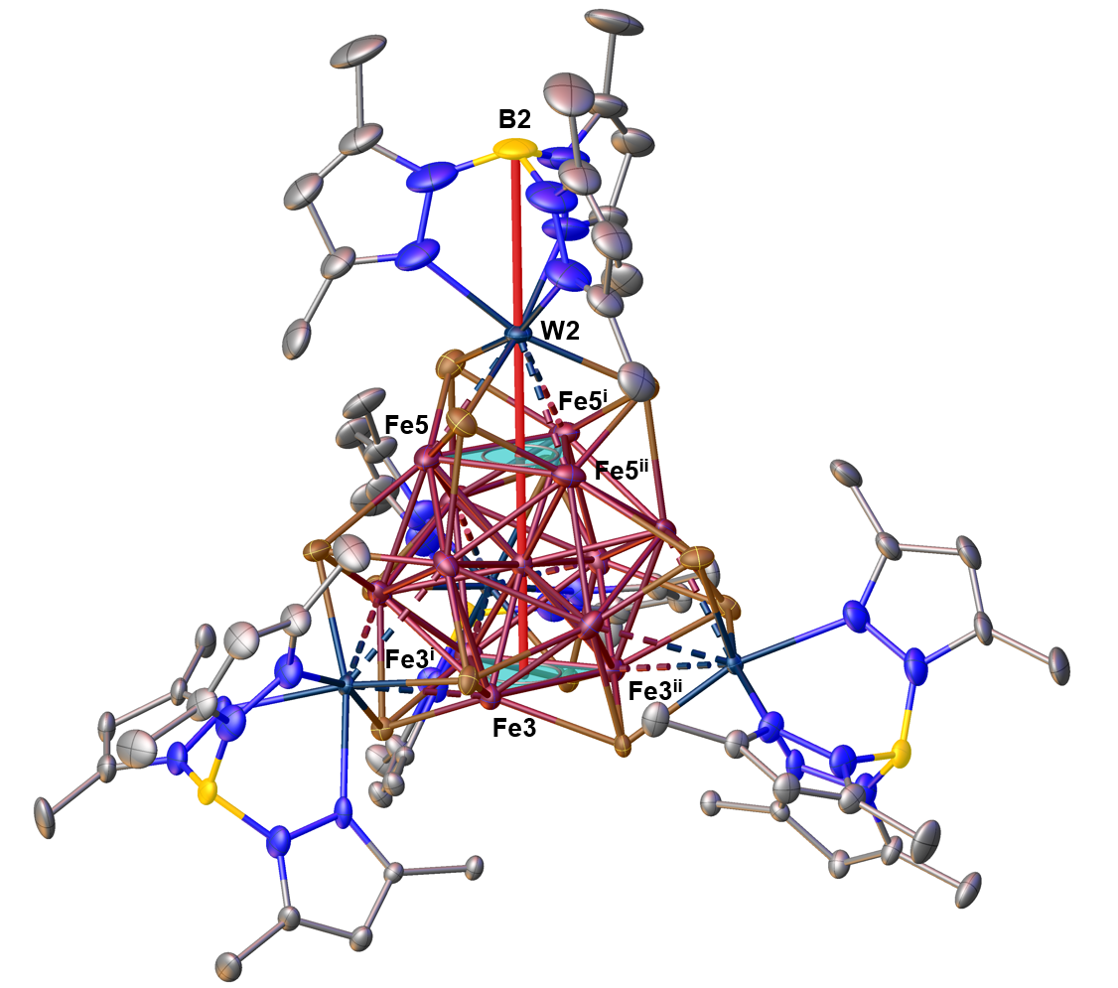


**Fig. S5.** The 3-fold axis in cluster **2**.


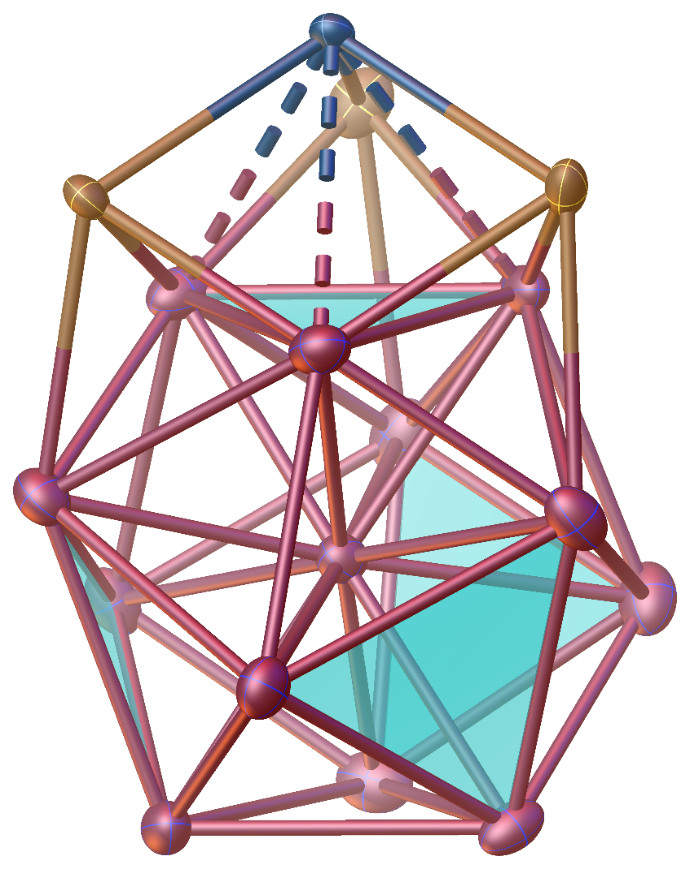


**Fig. S6.** Four of the triangles (Type **I**, as highlighted) are each covered by one [(Tp*)WS_3_] fragment. For clarity, only one [WS_3_] moiety is shown here, and the Tp* ligand has been omitted.


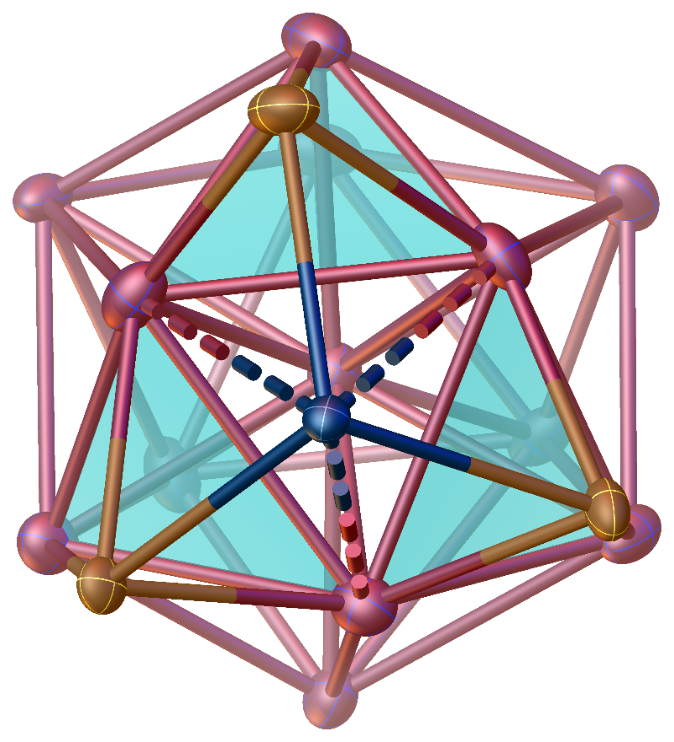


**Fig. S7.** Each sulfur atom of the [(Tp*)WS_3_] fragment covers a [Fe_3_] triangle (Type **II**, as highlighted). For clarity, only three of the twelve Type **II** [Fe_3_] triangles are highlighted. For clarity, only one [WS_3_] moiety is shown here with the Tp* ligand omitted, and all other [(Tp*)WS_3_] moieties are omitted.


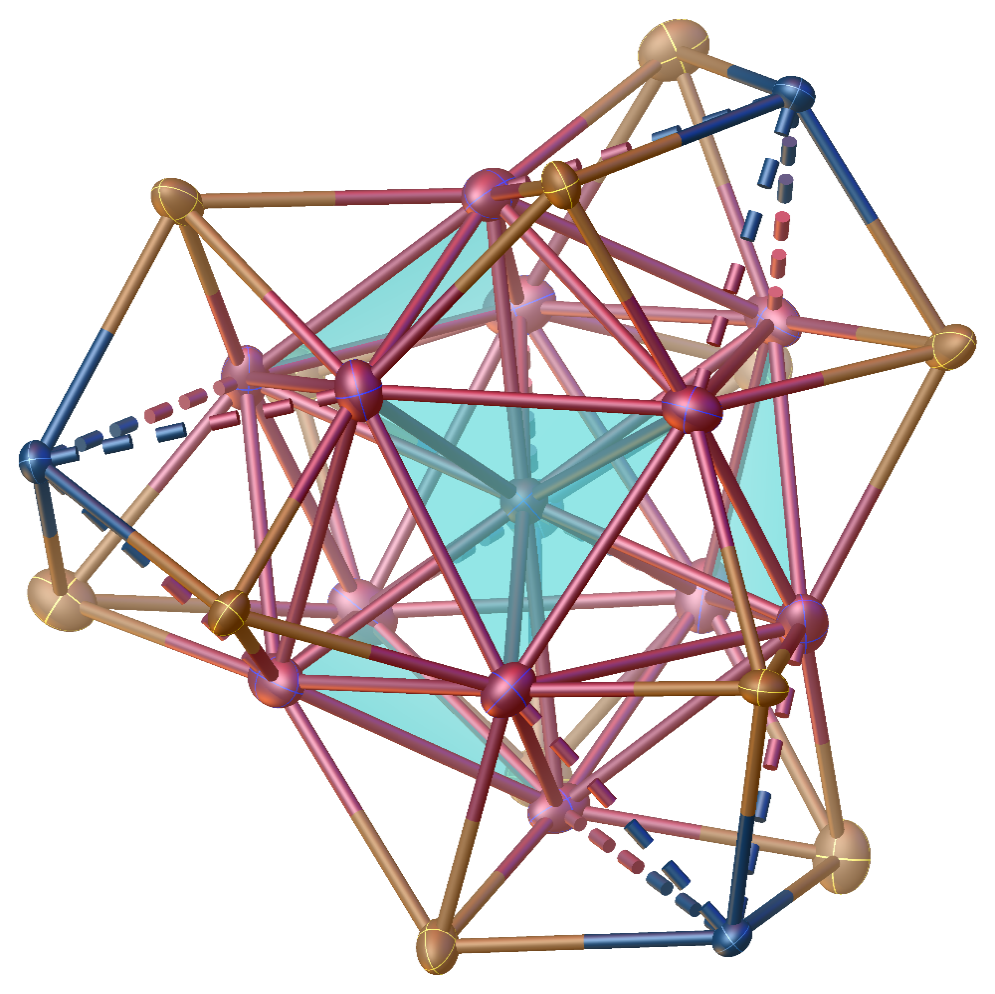


**Fig. S8.** There are four [Fe_3_] triangles (Type **III**, as highlighted) with no direct cover on top of them, exhibiting an open environment. For clarity, all Tp* ligands are omitted.

Crystallographic Data

## X-Ray Single-Crystal Diffraction

Single crystal of **1**•(BPh_4_) covered by APIEZON N Grease was mounted on a loop, and the diffraction measurement was carried out at 296.15 K on Bruker APEX II CCD diffractometer operating at 50 kV and 30 mA using Mo-Kα radiation (λ = 0.71073Å). Single crystal of **2**•(THF)_4.5_ mounted on a loop using Parabar 10312 oil was measured using a synchrotron radiation facility with SSRF BL-17B facilities (λ = 0.77488Å) at 100K. Data was collected by the strategy of multi-scan (φ and/or ω scans). All data were integrated by SANIT and scaled with either a numerical or multi-scan absorption correction using SADABS [11]. Structures were solved by SHELXT [12] and refined with SHELXL [13] using the OLEX2 [14] program. All non-hydrogen atoms were refined anisotropically, and all hydrogen atoms were added at idealized positions and refined using the riding model. X-ray crystallographic data have been deposited with the Cambridge Crystallographic Data Center (CCDC numbers 2106794 and 2106795).

## Crystallographic data for compounds

**Table S3.** Crystallographic data for compounds **1**•(BPh_4_) and **2**•(THF)_4.5_.

| Compound | **1**•(BPh_4_) | **2**•(THF)_4.5_ |
| --- | --- | --- |
| CCDC number | 2106795 | 2106794 |
| Empirical formula | C_57_H_87_B_2_ClFe_3_N_6_P_3_S_3_W | C_78_H_120.5_B_4_Fe_13_N_24_O_4.5_S_12_W_4_ |
| Formula weight | 1453.88 | 3355.89 |
| Temperature/K | 296.15 | 100 |
| Crystal system | monoclinic | trigonal |
| Space group | P2_1_/c | R$\bar{3}$ |
| a/Å | 11.534(8) | 18.5661(4) |
| b/Å | 15.651(11) | 18.5661(4) |
| c/Å | 36.46(3) | 60.8595(13) |
| α/° | 90 | 90 |
| β/° | 93.431(9) | 90 |
| γ/° | 90 | 120 |
| Volume/Å^3^ | 6570(8) | 18167.7(9) |
| Z | 4 | 6 |
| ρ_calc_g/cm^3^ | 1.47 | 1.84 |
| μ/mm^‑1^ | 2.643 | 6.931 |
| F(000) | 2972 | 9831 |
| Crystal size/mm^3^ | 0.12 × 0.1 × 0.05 | 0.04 × 0.03 × 0.03 |
| Radiation | MoKα (λ = 0.71073) | synchrotron (λ = 0.77488) |
| 2Θ range for data collection/° | 4.072 to 53.464 | 5.262 to 54.93 |
| Reflections collected | 49143 | 45622 |
| Independent reflections | 13923 [R_int_ = 0.0367] | 7063 [R_int_ = 0.0559] |
| Data/restraints/parameters | 13923/119/752 | 7063/214/497 |
| Goodness-of-fit on F^2^ | 1.184 | 1.043 |
| Final R indexes [I>=2σ (I)] | R_1_ = 0.0483, wR_2_ = 0.0836 | R_1_ = 0.0362, wR_2_ = 0.0917 |
| Final R indexes [all data] | R_1_ = 0.0656, wR_2_ = 0.0890 | R_1_ = 0.0433, wR_2_ = 0.0961 |
| Largest diff. peak/hole / e Å^-3^ | 1.14/-1.29 | 1.67/-1.34 |

^a^ R_1_ = Σ||F_o_|-|F_c_|| / Σ|F_o_|. ^b^ wR_2_ = [Σ[w(F_o_^2^-F_c_^2^)^2^] / Σ[w(F_o_^2^)^2^]]^1/2^.

**Table S4.** Bond lengths [Å] and angles [°] for **1**•(BPh4)

| W(1)-Fe(2) | 2.7005(17) | Fe(2)-S(3) | 2.2610(18) |
| --- | --- | --- | --- |
| W(1)-Fe(3) | 2.6890(19) | Fe(2)-Cl(1) | 2.475(2) |
| W(1)-Fe(1) | 2.6883(17) | Fe(2)-P(3) | 2.435(2) |
| W(1)-S(1) | 2.3746(16) | Fe(3)-Fe(1) | 2.5773(16) |
| W(1)-S(2) | 2.3928(17) | Fe(3)-S(1) | 2.2646(17) |
| W(1)-S(3) | 2.3734(17) | Fe(3)-S(3) | 2.2533(19) |
| W(1)-N(1) | 2.278(4) | Fe(3)-Cl(1) | 2.513(2) |
| W(1)-N(2) | 2.247(4) | Fe(3)-P(2) | 2.429(2) |
| W(1)-N(5) | 2.256(4) | Fe(1)-S(1) | 2.264(2) |
| Fe(2)-Fe(3) | 2.6152(17) | Fe(1)-S(2) | 2.2534(18) |
| Fe(2)-Fe(1) | 2.5988(17) | Fe(1)-Cl(1) | 2.500(2) |
| Fe(2)-S(2) | 2.264(2) |  |  |
|  |  |  |  |
| Fe(3)-W(1)-Fe(2) | 58.06(3) | S(3)-Fe(3)-Fe(2) | 54.73(4) |
| Fe(1)-W(1)-Fe(2) | 57.67(3) | S(3)-Fe(3)-Fe(1) | 104.35(7) |
| Fe(1)-W(1)-Fe(3) | 57.28(2) | S(3)-Fe(3)-S(1) | 110.56(6) |
| S(1)-W(1)-Fe(2) | 99.01(5) | S(3)-Fe(3)-Cl(1) | 107.07(7) |
| S(1)-W(1)-Fe(3) | 52.69(4) | S(3)-Fe(3)-P(2) | 121.97(6) |
| S(1)-W(1)-Fe(1) | 52.69(5) | Cl(1)-Fe(3)-W(1) | 108.82(4) |
| S(1)-W(1)-S(2) | 102.58(6) | Cl(1)-Fe(3)-Fe(2) | 57.67(5) |
| S(2)-W(1)-Fe(2) | 52.36(5) | Cl(1)-Fe(3)-Fe(1) | 58.80(5) |
| S(2)-W(1)-Fe(3) | 98.11(4) | P(2)-Fe(3)-W(1) | 162.48(5) |
| S(2)-W(1)-Fe(1) | 52.26(4) | P(2)-Fe(3)-Fe(2) | 134.43(5) |
| S(3)-W(1)-Fe(2) | 52.44(4) | P(2)-Fe(3)-Fe(1) | 130.02(6) |
| S(3)-W(1)-Fe(3) | 52.41(5) | P(2)-Fe(3)-Cl(1) | 88.48(6) |
| S(3)-W(1)-Fe(1) | 97.83(5) | Fe(2)-Fe(1)-W(1) | 61.40(5) |
| S(3)-W(1)-S(1) | 102.91(7) | Fe(3)-Fe(1)-W(1) | 61.37(5) |
| S(3)-W(1)-S(2) | 102.91(6) | Fe(3)-Fe(1)-Fe(2) | 60.70(4) |
| Fe(3)-Fe(2)-W(1) | 60.75(5) | S(1)-Fe(1)-W(1) | 56.52(4) |
| Fe(1)-Fe(2)-W(1) | 60.93(4) | S(1)-Fe(1)-Fe(2) | 105.07(6) |
| Fe(1)-Fe(2)-Fe(3) | 59.25(4) | S(1)-Fe(1)-Fe(3) | 55.32(4) |
| S(2)-Fe(2)-W(1) | 56.82(4) | S(1)-Fe(1)-Cl(1) | 109.94(7) |
| S(2)-Fe(2)-Fe(3) | 103.71(6) | S(1)-Fe(1)-P(1) | 117.18(6) |
| S(2)-Fe(2)-Fe(1) | 54.69(4) | S(2)-Fe(1)-W(1) | 57.11(5) |
| S(2)-Fe(2)-Cl(1) | 108.07(7) | S(2)-Fe(1)-Fe(2) | 55.07(5) |
| S(2)-Fe(2)-P(3) | 120.28(7) | S(2)-Fe(1)-Fe(3) | 105.23(7) |
| S(3)-Fe(2)-W(1) | 56.32(5) | S(2)-Fe(1)-S(1) | 110.87(6) |
| S(3)-Fe(2)-Fe(3) | 54.46(5) | S(2)-Fe(1)-Cl(1) | 107.58(7) |
| S(3)-Fe(2)-Fe(1) | 103.45(7) | S(2)-Fe(1)-P(1) | 118.06(7) |
| S(3)-Fe(2)-S(2) | 110.94(6) | Cl(1)-Fe(1)-W(1) | 109.25(5) |
| S(3)-Fe(2)-Cl(1) | 108.11(7) | Cl(1)-Fe(1)-Fe(2) | 58.05(4) |
| S(3)-Fe(2)-P(3) | 115.91(8) | Cl(1)-Fe(1)-Fe(3) | 59.32(5) |
| Cl(1)-Fe(2)-W(1) | 109.61(5) | P(1)-Fe(1)-W(1) | 160.05(5) |
| Cl(1)-Fe(2)-Fe(3) | 59.09(4) | P(1)-Fe(1)-Fe(2) | 134.49(6) |
| Cl(1)-Fe(2)-Fe(1) | 58.97(5) | P(1)-Fe(1)-Fe(3) | 133.56(6) |
| P(3)-Fe(2)-W(1) | 159.64(6) | P(1)-Fe(1)-Cl(1) | 90.70(6) |
| P(3)-Fe(2)-Fe(3) | 133.15(8) | Fe(3)-S(1)-W(1) | 70.80(6) |
| P(3)-Fe(2)-Fe(1) | 136.59(6) | Fe(1)-S(1)-W(1) | 70.79(5) |
| P(3)-Fe(2)-Cl(1) | 90.59(6) | Fe(1)-S(1)-Fe(3) | 69.37(6) |
| Fe(2)-Fe(3)-W(1) | 61.19(4) | Fe(2)-S(2)-W(1) | 70.83(5) |
| Fe(1)-Fe(3)-W(1) | 61.35(5) | Fe(1)-S(2)-W(1) | 70.63(5) |
| Fe(1)-Fe(3)-Fe(2) | 60.06(4) | Fe(1)-S(2)-Fe(2) | 70.24(7) |
| S(1)-Fe(3)-W(1) | 56.51(5) | Fe(2)-S(3)-W(1) | 71.23(6) |
| S(1)-Fe(3)-Fe(2) | 104.53(7) | Fe(3)-S(3)-W(1) | 71.01(6) |
| S(1)-Fe(3)-Fe(1) | 55.31(5) | Fe(3)-S(3)-Fe(2) | 70.80(6) |
| S(1)-Fe(3)-Cl(1) | 109.45(7) | Fe(2)-Cl(1)-Fe(3) | 63.24(5) |
| S(1)-Fe(3)-P(2) | 115.93(7) | Fe(2)-Cl(1)-Fe(1) | 62.99(5) |
| S(3)-Fe(3)-W(1) | 56.58(4) | Fe(1)-Cl(1)-Fe(3) | 61.88(4) |

**Table S5.** Bond lengths [Å] and angles [°] for **2**•(THF)4.5

| W(1)-Fe(2) | 2.7315(10) | Fe(1)-Fe(5)^ii^ | 2.4996(15) |
| --- | --- | --- | --- |
| W(1)-Fe(3) | 2.6982(9) | Fe(1)-Fe(5) | 2.4996(15) |
| W(1)-Fe(4) | 2.6977(9) | Fe(1)-Fe(5)^I^ | 2.4995(15) |
| W(1)-S(1) | 2.3745(14) | Fe(2)-Fe(3)^i^ | 2.3774(14) |
| W(1)-S(2) | 2.3908(14) | Fe(2)-Fe(3) | 2.7925(13) |
| W(1)-S(3) | 2.3912(16) | Fe(2)-Fe(4)^i^ | 2.6930(13) |
| W(1)-N(1) | 2.263(5) | Fe(2)-Fe(4) | 2.6864(14) |
| W(1)-N(2) | 2.258(5) | Fe(2)-Fe(5) | 2.5891(14) |
| W(1)-N(6) | 2.247(5) | Fe(2)-S(1) | 2.3126(17) |
| W(2)-Fe(5) | 2.6910(11) | Fe(2)-S(2)^i^ | 2.5654(19) |
| W(2)-Fe(5)^i^ | 2.6910(11) | Fe(2)-S(3) | 2.218(2) |
| W(2)-Fe(5)^ii^ | 2.6911(11) | Fe(3)-Fe(3)^ii^ | 2.6738(17) |
| W(2)-S(4)^ii^ | 2.4000(19) | Fe(3)-Fe(3)^i^ | 2.6738(16) |
| W(2)-S(4)^i^ | 2.4000(18) | Fe(3)-Fe(4) | 2.7228(13) |
| W(2)-S(4) | 2.4000(18) | Fe(3)-S(1)^ii^ | 2.4381(16) |
| W(2)-N(7)^ii^ | 2.232(6) | Fe(3)-S(1) | 2.2064(17) |
| W(2)-N(7) | 2.232(6) | Fe(3)-S(2) | 2.2840(18) |
| W(2)-N(7)^i^ | 2.232(6) | Fe(4)-Fe(5)^ii^ | 2.6175(14) |
| Fe(1)-Fe(2)^ii^ | 2.4994(10) | Fe(4)-Fe(5) | 2.4579(15) |
| Fe(1)-Fe(2)^i^ | 2.4994(10) | Fe(4)-S(2) | 2.233(2) |
| Fe(1)-Fe(2) | 2.4994(10) | Fe(4)-S(3) | 2.300(2) |
| Fe(1)-Fe(3) | 2.4832(15) | Fe(4)-S(4)^ii^ | 2.499(2) |
| Fe(1)-Fe(3)^i^ | 2.4834(15) | Fe(5)-Fe(5)^i^ | 2.7429(18) |
| Fe(1)-Fe(3)^ii^ | 2.4834(15) | Fe(5)-Fe(5)^ii^ | 2.7430(18) |
| Fe(1)-Fe(4)^ii^ | 2.5301(9) | Fe(5)-S(3) | 2.683(2) |
| Fe(1)-Fe(4) | 2.5301(10) | Fe(5)-S(4)^ii^ | 2.279(2) |
| Fe(1)-Fe(4)^i^ | 2.5301(9) | Fe(5)-S(4) | 2.222(2) |
|  |  |  |  |
| Fe(3)-W(1)-Fe(2) | 61.90(3) | S(3)-Fe(2)-S(2)^i^ | 140.41(7) |
| Fe(4)-W(1)-Fe(2) | 59.31(3) | W(1)-Fe(3)-Fe(2) | 59.64(3) |
| Fe(4)-W(1)-Fe(3) | 60.61(3) | W(1)-Fe(3)-Fe(4) | 59.69(3) |
| S(1)-W(1)-Fe(2) | 53.31(4) | Fe(1)-Fe(3)-W(1) | 106.15(4) |
| S(1)-W(1)-Fe(3) | 51.08(4) | Fe(1)-Fe(3)-Fe(2) | 56.19(3) |
| S(1)-W(1)-Fe(4) | 98.67(4) | Fe(1)-Fe(3)-Fe(3)^ii^ | 57.43(2) |
| S(1)-W(1)-S(2) | 102.79(5) | Fe(1)-Fe(3)-Fe(3)^i^ | 57.43(2) |
| S(1)-W(1)-S(3) | 102.49(6) | Fe(1)-Fe(3)-Fe(4) | 57.94(4) |
| S(2)-W(1)-Fe(2) | 100.14(5) | Fe(2)^ii^-Fe(3)-W(1) | 116.05(4) |
| S(2)-W(1)-Fe(3) | 52.92(4) | Fe(2)^ii^-Fe(3)-Fe(1) | 61.85(3) |
| S(2)-W(1)-Fe(4) | 51.62(5) | Fe(2)^ii^-Fe(3)-Fe(2) | 109.95(5) |
| S(2)-W(1)-S(3) | 102.98(6) | Fe(2)^ii^-Fe(3)-Fe(3)^i^ | 113.69(4) |
| S(3)-W(1)-Fe(2) | 50.79(5) | Fe(2)^ii^-Fe(3)-Fe(3)^ii^ | 66.83(4) |
| S(3)-W(1)-Fe(3) | 101.36(5) | Fe(2)^ii^-Fe(3)-Fe(4) | 63.32(4) |
| S(3)-W(1)-Fe(4) | 53.32(5) | Fe(2)^ii^-Fe(3)-S(1)^ii^ | 57.38(5) |
| Fe(5)^i^-W(2)-Fe(5) | 61.28(4) | Fe(3)^i^-Fe(3)-W(1) | 104.15(5) |
| Fe(5)^i^-W(2)-Fe(5)^ii^ | 61.28(4) | Fe(3)^ii^-Fe(3)-W(1) | 161.03(4) |
| Fe(5)-W(2)-Fe(5)^ii^ | 61.28(4) | Fe(3)^i^-Fe(3)-Fe(2) | 51.51(4) |
| S(4)-W(2)-Fe(5)^ii^ | 100.48(5) | Fe(3)^ii^-Fe(3)-Fe(2) | 101.44(4) |
| S(4)^i^-W(2)-Fe(5) | 100.47(5) | Fe(3)^i^-Fe(3)-Fe(3)^ii^ | 60 |
| S(4)^ii^-W(2)-Fe(5)^ii^ | 51.38(6) | Fe(3)^ii^-Fe(3)-Fe(4) | 110.79(3) |
| S(4)^i^-W(2)-Fe(5)^i^ | 51.38(6) | Fe(3)^i^-Fe(3)-Fe(4) | 101.85(4) |
| S(4)^ii^-W(2)-Fe(5) | 52.82(5) | Fe(4)-Fe(3)-Fe(2) | 58.28(4) |
| S(4)-W(2)-Fe(5) | 51.38(6) | S(1)-Fe(3)-W(1) | 56.86(4) |
| S(4)-W(2)-Fe(5)^i^ | 52.82(5) | S(1)^ii^-Fe(3)-W(1) | 147.58(5) |
| S(4)^ii^-W(2)-Fe(5)^i^ | 100.47(5) | S(1)^ii^-Fe(3)-Fe(1) | 97.66(5) |
| S(4)^i^-W(2)-Fe(5)^ii^ | 52.82(5) | S(1)-Fe(3)-Fe(1) | 104.22(5) |
| S(4)^ii^-W(2)-S(4)^i^ | 102.75(5) | S(1)-Fe(3)-Fe(2)^ii^ | 163.46(6) |
| S(4)^ii^-W(2)-S(4) | 102.75(5) | S(1)^ii^-Fe(3)-Fe(2) | 151.62(6) |
| S(4)^i^-W(2)-S(4) | 102.74(5) | S(1)-Fe(3)-Fe(2) | 53.57(5) |
| N(7)-W(2)-Fe(5)^i^ | 137.76(18) | S(1)-Fe(3)-Fe(3)^ii^ | 114.66(5) |
| N(7)^ii^-W(2)-Fe(5) | 137.76(19) | S(1)^ii^-Fe(3)-Fe(3)^i^ | 107.16(5) |
| N(7)-W(2)-Fe(5)^ii^ | 138.60(19) | S(1)-Fe(3)-Fe(3)^i^ | 59.02(5) |
| N(7)^i^-W(2)-Fe(5)^i^ | 93.98(19) | S(1)^ii^-Fe(3)-Fe(3)^ii^ | 50.88(5) |
| N(7)^i^-W(2)-Fe(5) | 138.60(19) | S(1)^ii^-Fe(3)-Fe(4) | 120.27(5) |
| N(7)^ii^-W(2)-Fe(5)^ii^ | 93.98(18) | S(1)-Fe(3)-Fe(4) | 102.33(5) |
| N(7)^i^-W(2)-Fe(5)^ii^ | 137.76(18) | S(1)-Fe(3)-S(1)^ii^ | 137.38(8) |
| N(7)-W(2)-Fe(5) | 93.97(18) | S(1)-Fe(3)-S(2) | 112.04(6) |
| N(7)^ii^-W(2)-Fe(5)^i^ | 138.60(19) | S(2)-Fe(3)-W(1) | 56.62(4) |
| N(7)^ii^-W(2)-S(4) | 165.45(19) | S(2)-Fe(3)-Fe(1) | 105.41(6) |
| N(7)-W(2)-S(4)^ii^ | 87.3(2) | S(2)-Fe(3)-Fe(2) | 101.12(6) |
| N(7)^i^-W(2)-S(4)^i^ | 84.95(19) | S(2)-Fe(3)-Fe(2)^ii^ | 66.75(5) |
| N(7)^ii^-W(2)-S(4)^i^ | 87.3(2) | S(2)-Fe(3)-Fe(3)^ii^ | 132.91(6) |
| N(7)-W(2)-S(4)^i^ | 165.46(19) | S(2)-Fe(3)-Fe(3)^i^ | 152.11(6) |
| N(7)-W(2)-S(4) | 84.95(19) | S(2)-Fe(3)-Fe(4) | 52.06(5) |
| N(7)^ii^-W(2)-S(4)^ii^ | 84.95(19) | S(2)-Fe(3)-S(1)^ii^ | 96.30(6) |
| N(7)^i^-W(2)-S(4)^ii^ | 165.45(19) | W(1)-Fe(4)-Fe(3) | 59.70(3) |
| N(7)^i^-W(2)-S(4) | 87.3(2) | Fe(1)-Fe(4)-W(1) | 104.83(4) |
| N(7)^i^-W(2)-N(7)^ii^ | 83.1(3) | Fe(1)-Fe(4)-Fe(2)^ii^ | 57.07(3) |
| N(7)-W(2)-N(7)^ii^ | 83.1(3) | Fe(1)-Fe(4)-Fe(2) | 57.16(3) |
| N(7)-W(2)-N(7)^i^ | 83.1(3) | Fe(1)-Fe(4)-Fe(3) | 56.28(4) |
| Fe(2)^i^-Fe(1)-Fe(2)^ii^ | 116.04(3) | Fe(1)-Fe(4)-Fe(5)^ii^ | 58.07(4) |
| Fe(2)^i^-Fe(1)-Fe(2) | 116.04(3) | Fe(2)-Fe(4)-W(1) | 60.97(3) |
| Fe(2)-Fe(1)-Fe(2)^ii^ | 116.04(3) | Fe(2)^ii^-Fe(4)-W(1) | 106.12(4) |
| Fe(2)^i^-Fe(1)-Fe(4) | 177.93(7) | Fe(2)-Fe(4)-Fe(2)^ii^ | 104.04(5) |
| Fe(2)^ii^-Fe(1)-Fe(4)^ii^ | 64.57(3) | Fe(2)-Fe(4)-Fe(3) | 62.16(3) |
| Fe(2)-Fe(1)-Fe(4) | 64.57(3) | Fe(2)^ii^-Fe(4)-Fe(3) | 52.07(3) |
| Fe(2)-Fe(1)-Fe(4)^ii^ | 177.94(7) | Fe(5)^ii^-Fe(4)-W(1) | 160.78(5) |
| Fe(2)^i^-Fe(1)-Fe(4)^ii^ | 64.75(3) | Fe(5)-Fe(4)-W(1) | 115.94(4) |
| Fe(2)^i^-Fe(1)-Fe(4)^i^ | 64.57(3) | Fe(5)-Fe(4)-Fe(1) | 60.13(4) |
| Fe(2)-Fe(1)-Fe(4)^i^ | 64.74(3) | Fe(5)-Fe(4)-Fe(2) | 60.24(4) |
| Fe(2)^ii^-Fe(1)-Fe(4)^i^ | 177.94(7) | Fe(5)^ii^-Fe(4)-Fe(2) | 109.26(4) |
| Fe(2)^ii^-Fe(1)-Fe(4) | 64.75(3) | Fe(5)-Fe(4)-Fe(2)^ii^ | 110.47(5) |
| Fe(2)-Fe(1)-Fe(5) | 62.39(4) | Fe(5)^ii^-Fe(4)-Fe(2)^ii^ | 58.34(4) |
| Fe(2)^ii^-Fe(1)-Fe(5)^i^ | 119.81(5) | Fe(5)^ii^-Fe(4)-Fe(3) | 101.30(4) |
| Fe(2)^i^-Fe(1)-Fe(5) | 119.81(5) | Fe(5)-Fe(4)-Fe(3) | 109.65(5) |
| Fe(2)^i^-Fe(1)-Fe(5)^ii^ | 115.75(5) | Fe(5)-Fe(4)-Fe(5)^ii^ | 65.34(5) |
| Fe(2)^ii^-Fe(1)-Fe(5)^ii^ | 62.39(4) | Fe(5)-Fe(4)-S(4)^ii^ | 54.74(5) |
| Fe(2)-Fe(1)-Fe(5)^i^ | 115.75(5) | S(2)-Fe(4)-W(1) | 57.08(4) |
| Fe(2)-Fe(1)-Fe(5)^ii^ | 119.81(5) | S(2)-Fe(4)-Fe(1) | 105.46(6) |
| Fe(2)^ii^-Fe(1)-Fe(5) | 115.75(5) | S(2)-Fe(4)-Fe(2)^ii^ | 61.95(5) |
| Fe(2)^i^-Fe(1)-Fe(5)^i^ | 62.39(4) | S(2)-Fe(4)-Fe(2) | 105.84(5) |
| Fe(3)^ii^-Fe(1)-Fe(2)^ii^ | 68.17(4) | S(2)-Fe(4)-Fe(3) | 53.80(5) |
| Fe(3)-Fe(1)-Fe(2) | 68.17(4) | S(2)-Fe(4)-Fe(5) | 163.40(6) |
| Fe(3)^ii^-Fe(1)-Fe(2) | 116.27(6) | S(2)-Fe(4)-Fe(5)^ii^ | 115.75(6) |
| Fe(3)^ii^-Fe(1)-Fe(2)^i^ | 56.99(4) | S(2)-Fe(4)-S(3) | 111.30(7) |
| Fe(3)-Fe(1)-Fe(2)^i^ | 116.28(6) | S(2)-Fe(4)-S(4)^ii^ | 140.12(7) |
| Fe(3)^i^-Fe(1)-Fe(2) | 57.00(4) | S(3)-Fe(4)-W(1) | 56.50(5) |
| Fe(3)^i^-Fe(1)-Fe(2)^i^ | 68.17(4) | S(3)-Fe(4)-Fe(1) | 106.02(6) |
| Fe(3)^i^-Fe(1)-Fe(2)^ii^ | 116.27(6) | S(3)-Fe(4)-Fe(2)^ii^ | 154.33(6) |
| Fe(3)-Fe(1)-Fe(2)^ii^ | 56.99(4) | S(3)-Fe(4)-Fe(2) | 52.13(6) |
| Fe(3)^i^-Fe(1)-Fe(3)^ii^ | 65.14(5) | S(3)-Fe(4)-Fe(3) | 103.10(6) |
| Fe(3)-Fe(1)-Fe(3)^i^ | 65.14(5) | S(3)-Fe(4)-Fe(5) | 68.57(6) |
| Fe(3)-Fe(1)-Fe(3)^ii^ | 65.14(5) | S(3)-Fe(4)-Fe(5)^ii^ | 132.79(7) |
| Fe(3)^ii^-Fe(1)-Fe(4)^ii^ | 65.78(3) | S(3)-Fe(4)-S(4)^ii^ | 93.19(7) |
| Fe(3)^i^-Fe(1)-Fe(4)^ii^ | 124.74(5) | S(4)^ii^-Fe(4)-W(1) | 146.45(6) |
| Fe(3)-Fe(1)-Fe(4) | 65.79(3) | S(4)^ii^-Fe(4)-Fe(1) | 96.76(6) |
| Fe(3)-Fe(1)-Fe(4)^i^ | 124.74(5) | S(4)^ii^-Fe(4)-Fe(2)^ii^ | 107.14(6) |
| Fe(3)^ii^-Fe(1)-Fe(4)^i^ | 113.37(4) | S(4)^ii^-Fe(4)-Fe(2) | 114.03(6) |
| Fe(3)^ii^-Fe(1)-Fe(4) | 124.75(5) | S(4)^ii^-Fe(4)-Fe(3) | 151.32(6) |
| Fe(3)-Fe(1)-Fe(4)^ii^ | 113.37(4) | S(4)^ii^-Fe(4)-Fe(5)^ii^ | 51.42(5) |
| Fe(3)^i^-Fe(1)-Fe(4)^i^ | 65.78(3) | W(2)-Fe(5)-Fe(5)^ii^ | 59.361(18) |
| Fe(3)^i^-Fe(1)-Fe(4) | 113.37(4) | W(2)-Fe(5)-Fe(5)^i^ | 59.360(18) |
| Fe(3)^ii^-Fe(1)-Fe(5)^ii^ | 116.51(3) | Fe(1)-Fe(5)-W(2) | 104.64(4) |
| Fe(3)^i^-Fe(1)-Fe(5) | 111.97(3) | Fe(1)-Fe(5)-Fe(2) | 58.80(4) |
| Fe(3)^ii^-Fe(1)-Fe(5) | 176.08(4) | Fe(1)-Fe(5)-Fe(4)^i^ | 59.21(3) |
| Fe(3)-Fe(1)-Fe(5)^i^ | 176.08(4) | Fe(1)-Fe(5)-Fe(5)^i^ | 56.72(3) |
| Fe(3)^i^-Fe(1)-Fe(5)^ii^ | 176.08(4) | Fe(1)-Fe(5)-Fe(5)^ii^ | 56.72(3) |
| Fe(3)^ii^-Fe(1)-Fe(5)^i^ | 111.97(3) | Fe(1)-Fe(5)-S(3) | 96.22(5) |
| Fe(3)-Fe(1)-Fe(5)^ii^ | 111.96(3) | Fe(2)-Fe(5)-W(2) | 163.10(5) |
| Fe(3)^i^-Fe(1)-Fe(5)^i^ | 116.52(3) | Fe(2)-Fe(5)-Fe(4)^i^ | 62.29(4) |
| Fe(3)-Fe(1)-Fe(5) | 116.51(3) | Fe(2)-Fe(5)-Fe(5)^i^ | 105.08(4) |
| Fe(4)^ii^-Fe(1)-Fe(4)^i^ | 114.58(3) | Fe(2)-Fe(5)-Fe(5)^ii^ | 108.38(3) |
| Fe(4)-Fe(1)-Fe(4)^i^ | 114.58(3) | Fe(2)-Fe(5)-S(3) | 49.72(5) |
| Fe(4)-Fe(1)-Fe(4)^ii^ | 114.58(3) | Fe(4)-Fe(5)-W(2) | 112.60(4) |
| Fe(5)^ii^-Fe(1)-Fe(4)^i^ | 115.55(6) | Fe(4)^i^-Fe(5)-W(2) | 107.66(5) |
| Fe(5)-Fe(1)-Fe(4)^i^ | 62.72(4) | Fe(4)-Fe(5)-Fe(1) | 61.37(3) |
| Fe(5)-Fe(1)-Fe(4)^ii^ | 115.55(6) | Fe(4)-Fe(5)-Fe(2) | 64.26(4) |
| Fe(5)^ii^-Fe(1)-Fe(4) | 62.72(4) | Fe(4)-Fe(5)-Fe(4)^i^ | 114.01(5) |
| Fe(5)^i^-Fe(1)-Fe(4)^ii^ | 62.72(4) | Fe(4)^i^-Fe(5)-Fe(5)^ii^ | 105.05(4) |
| Fe(5)^ii^-Fe(1)-Fe(4)^ii^ | 58.51(4) | Fe(4)-Fe(5)-Fe(5)^i^ | 109.67(4) |
| Fe(5)^i^-Fe(1)-Fe(4)^i^ | 58.51(4) | Fe(4)-Fe(5)-Fe(5)^ii^ | 60.14(5) |
| Fe(5)-Fe(1)-Fe(4) | 58.50(4) | Fe(4)^i^-Fe(5)-Fe(5)^i^ | 54.53(5) |
| Fe(5)^i^-Fe(1)-Fe(4) | 115.54(6) | Fe(4)-Fe(5)-S(3) | 52.92(5) |
| Fe(5)^i^-Fe(1)-Fe(5) | 66.55(5) | Fe(4)^i^-Fe(5)-S(3) | 109.05(6) |
| Fe(5)-Fe(1)-Fe(5)^ii^ | 66.55(5) | Fe(5)^i^-Fe(5)-Fe(5)^ii^ | 60 |
| Fe(5)^i^-Fe(1)-Fe(5)^ii^ | 66.55(5) | S(3)-Fe(5)-W(2) | 143.20(6) |
| W(1)-Fe(2)-Fe(3) | 58.46(3) | S(3)-Fe(5)-Fe(5)^ii^ | 112.34(7) |
| Fe(1)-Fe(2)-W(1) | 104.70(3) | S(3)-Fe(5)-Fe(5)^i^ | 152.35(4) |
| Fe(1)-Fe(2)-Fe(3) | 55.64(4) | S(4)^ii^-Fe(5)-W(2) | 57.02(5) |
| Fe(1)-Fe(2)-Fe(4)^i^ | 58.18(3) | S(4)-Fe(5)-W(2) | 57.53(5) |
| Fe(1)-Fe(2)-Fe(4) | 58.27(3) | S(4)^ii^-Fe(5)-Fe(1) | 103.67(7) |
| Fe(1)-Fe(2)-Fe(5) | 58.81(5) | S(4)-Fe(5)-Fe(1) | 105.39(6) |
| Fe(1)-Fe(2)-S(2)^i^ | 97.03(5) | S(4)^ii^-Fe(5)-Fe(2) | 126.58(7) |
| Fe(3)^i^-Fe(2)-W(1) | 111.89(4) | S(4)-Fe(5)-Fe(2) | 120.39(7) |
| Fe(3)^i^-Fe(2)-Fe(1) | 61.16(4) | S(4)^ii^-Fe(5)-Fe(4)^i^ | 155.58(7) |
| Fe(3)^i^-Fe(2)-Fe(3) | 61.67(5) | S(4)^ii^-Fe(5)-Fe(4) | 63.55(6) |
| Fe(3)^i^-Fe(2)-Fe(4)^i^ | 64.61(4) | S(4)-Fe(5)-Fe(4) | 162.63(7) |
| Fe(3)^i^-Fe(2)-Fe(4) | 111.51(5) | S(4)-Fe(5)-Fe(4)^i^ | 61.54(6) |
| Fe(3)^i^-Fe(2)-Fe(5) | 112.46(5) | S(4)-Fe(5)-Fe(5)^ii^ | 103.70(6) |
| Fe(3)^i^-Fe(2)-S(2)^i^ | 54.89(5) | S(4)^ii^-Fe(5)-Fe(5)^ii^ | 51.52(7) |
| Fe(4)^i^-Fe(2)-W(1) | 162.57(4) | S(4)^ii^-Fe(5)-Fe(5)^i^ | 102.15(6) |
| Fe(4)-Fe(2)-W(1) | 59.72(3) | S(4)-Fe(5)-Fe(5)^i^ | 53.41(6) |
| Fe(4)^i^-Fe(2)-Fe(3) | 108.12(4) | S(4)-Fe(5)-S(3) | 143.90(8) |
| Fe(4)-Fe(2)-Fe(3) | 59.56(3) | S(4)^ii^-Fe(5)-S(3) | 88.96(7) |
| Fe(4)-Fe(2)-Fe(4)^i^ | 104.65(5) | S(4)-Fe(5)-S(4)^ii^ | 112.81(10) |
| Fe(5)-Fe(2)-W(1) | 110.51(5) | W(1)-S(1)-Fe(3)^i^ | 123.40(6) |
| Fe(5)-Fe(2)-Fe(3) | 103.82(4) | Fe(2)-S(1)-W(1) | 71.28(5) |
| Fe(5)-Fe(2)-Fe(4) | 55.50(4) | Fe(2)-S(1)-Fe(3)^i^ | 59.99(5) |
| Fe(5)-Fe(2)-Fe(4)^i^ | 59.37(4) | Fe(3)-S(1)-W(1) | 72.07(5) |
| S(1)-Fe(2)-W(1) | 55.42(4) | Fe(3)-S(1)-Fe(2) | 76.29(6) |
| S(1)-Fe(2)-Fe(1) | 100.62(6) | Fe(3)-S(1)-Fe(3)^i^ | 70.09(6) |
| S(1)-Fe(2)-Fe(3) | 50.14(4) | W(1)-S(2)-Fe(2)^ii^ | 120.73(7) |
| S(1)-Fe(2)-Fe(3)^i^ | 62.62(5) | Fe(3)-S(2)-W(1) | 70.46(5) |
| S(1)-Fe(2)-Fe(4)^i^ | 126.73(6) | Fe(3)-S(2)-Fe(2)^ii^ | 58.36(5) |
| S(1)-Fe(2)-Fe(4) | 100.59(5) | Fe(4)-S(2)-W(1) | 71.30(5) |
| S(1)-Fe(2)-Fe(5) | 153.59(6) | Fe(4)-S(2)-Fe(2)^ii^ | 67.88(6) |
| S(1)-Fe(2)-S(2)^i^ | 92.19(6) | Fe(4)-S(2)-Fe(3) | 74.14(6) |
| S(2)^i^-Fe(2)-W(1) | 143.44(5) | W(1)-S(3)-Fe(5) | 118.90(7) |
| S(2)^i^-Fe(2)-Fe(3) | 116.09(5) | Fe(2)-S(3)-W(1) | 72.57(5) |
| S(2)^i^-Fe(2)-Fe(4)^i^ | 50.17(4) | Fe(2)-S(3)-Fe(4) | 72.95(6) |
| S(2)^i^-Fe(2)-Fe(4) | 153.75(6) | Fe(2)-S(3)-Fe(5) | 62.93(6) |
| S(2)^i^-Fe(2)-Fe(5) | 105.85(5) | Fe(4)-S(3)-W(1) | 70.18(5) |
| S(3)-Fe(2)-W(1) | 56.64(5) | Fe(4)-S(3)-Fe(5) | 58.51(5) |
| S(3)-Fe(2)-Fe(1) | 109.68(7) | W(2)-S(4)-Fe(4)^i^ | 122.02(8) |
| S(3)-Fe(2)-Fe(3) | 103.17(6) | Fe(5)-S(4)-W(2) | 71.09(6) |
| S(3)-Fe(2)-Fe(3)^i^ | 164.70(7) | Fe(5)^i^-S(4)-W(2) | 70.16(6) |
| S(3)-Fe(2)-Fe(4)^i^ | 122.76(6) | Fe(5)^i^-S(4)-Fe(4)^i^ | 61.71(6) |
| S(3)-Fe(2)-Fe(4) | 54.92(5) | Fe(5)-S(4)-Fe(4)^i^ | 67.04(6) |
| S(3)-Fe(2)-Fe(5) | 67.35(6) | Fe(5)-S(4)-Fe(5)^i^ | 75.07(8) |
| S(3)-Fe(2)-S(1) | 110.21(7) |  |  |

Symmetry transformations used to generate equivalent atoms: ^i^ -y,x-y,z ^ii^ -x+y,-x,z

Theoretical and Computational Section

## 1 Computational methods

The geometry optimization of **2-H** at various spins (S=26/2 to S=36/2) was carried out using Amsterdam Density Functional program (ADF 2019.304) [15] with the PBE [16] functional and the Slater-type orbital (STO) basis sets of triple-zeta quality plus polarization functions (TZP) [17] for Fe and W and double-zeta quality plus polarization functions (DZP) [17] for H, B, C, N, S atoms. Scalar relativistic (SR) effects were taken into consideration through the zero-order-regular approximation (ZORA) Hamiltonian [18].

The geometry optimization of **2** at S=32/2 was carried out at the DFT level of theory as implemented in the Gaussian 16 program [19]. Various functionals (BP86 [20, 21], PBE [16], BLYP [20, 22], TPSS [23-25], B3LYP [26], PBE0 [27], M06 [28] were used in conjunction with Lanl2DZ basis sets and the associated pseudopotentials for Fe, W [29] and 6-31G* basis sets for other elements. The geometry of **2-H** for bonding analysis was obtained from geometry optimization at the PBE/def2-SVP level and T point group symmetry. Vibrational frequency calculation was performed to confirm the presented structure has no imaginary frequency. Spin natural orbitals (SNOs) and Pipek-Mezey localized molecular orbitals (PM-LMOs) were calculated using Multiwfn program [30]. Principal interacting spin orbital (PISO) analysis was carried out and corresponding natural atomic orbitals (NAOs) were obtained with NBO 7.0 software [31]. The composition of Pipek-Mezey LMOs and bonding PIMOs were calculated with Hirshfeld scheme using Multiwfn program.

## 2 Model structures for calculations

Two models were considered in this work, including experimental structure **2** and a simplified model **2-H** (**Fig. S9**). The simplification of **2** (205 atoms, C_3_ point group) was to replace all methyl groups with hydrogen atoms, leading to **2-H** (133 atoms, T point group). The aim of model simplification is to save time for the calculations as well as enable applying high symmetry to better understand the electronic structures. Functionals were tested using **2** while the bonding analysis was performed based on the **2-H** model.

**2**


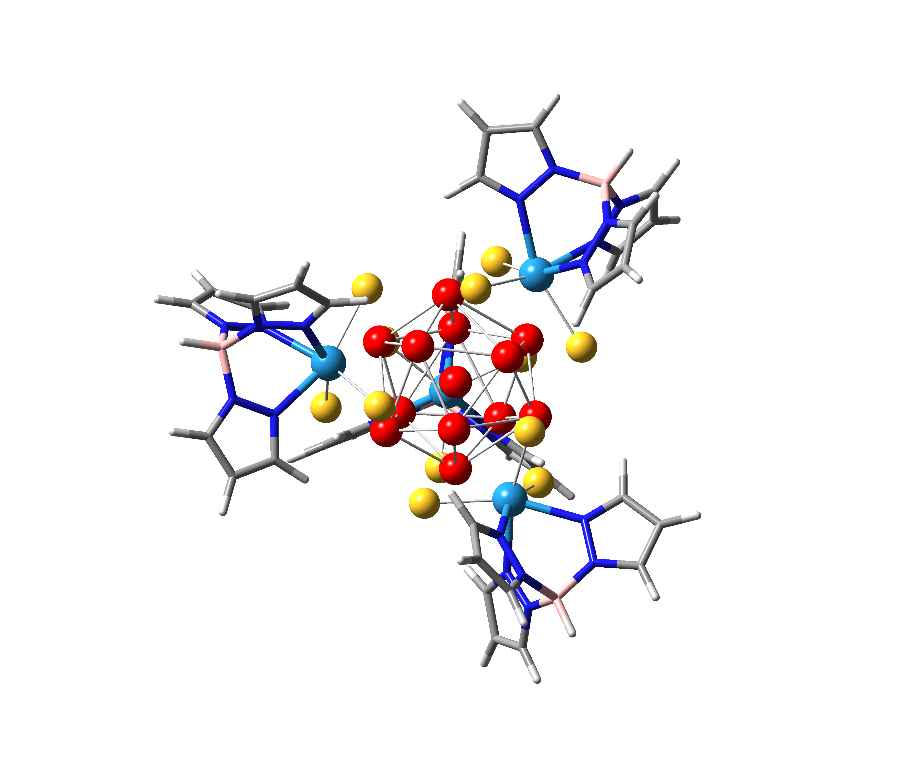

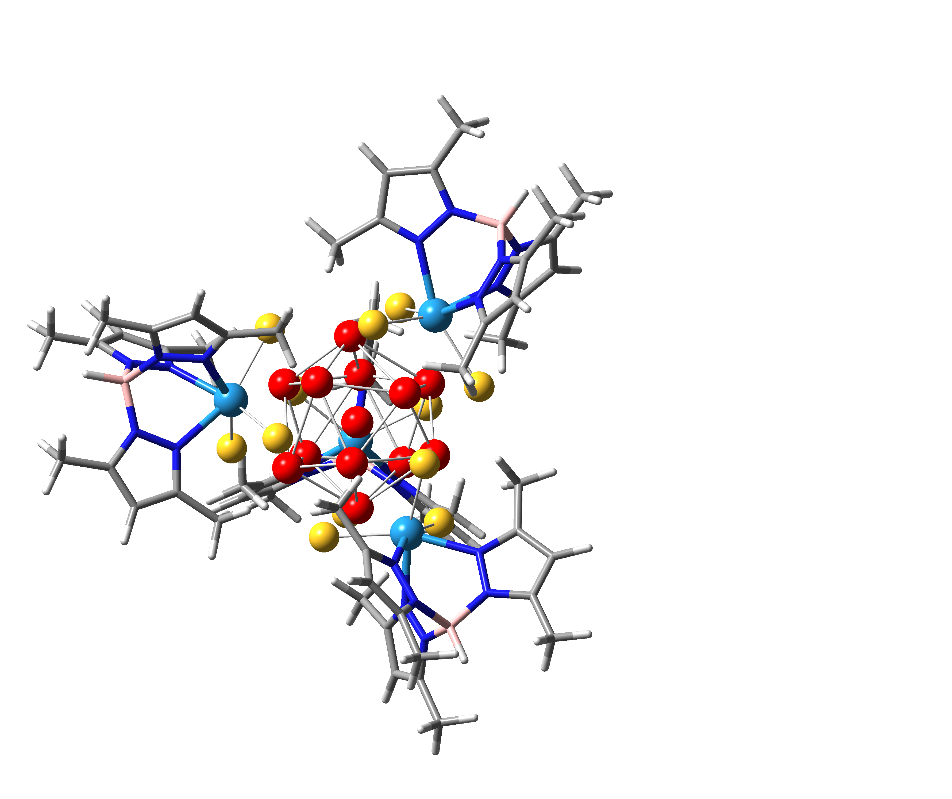


**2-H**

**Fig. S9.** Crystal structure of **2** and the optimized structure of the simplified model **2-H** at the PBE/def2-SVP level.

## 3 Relative energies for different spin states

To computationally predict the ground spin state, we performed structure optimization of **2-H** at various spins and compared the corresponding energy values and confirmed the S=32/2 to be the lowest spin state (**Fig. S10**). We noted a paper on a similar structure (*Angew. Chem. Int. Ed*. 2023, e202313880) published online after our submission. In this paper the ground state was computationally predicted as S=32/2 (=16), consistent with ours. However, they assigned spin S = 26/2 (=13) based on direct current (dc) susceptibility measurements. They claimed that the energies of both the S=16 and S=13 spin states are very close and EPR simulations under both spin states can give reasonable results. Therefore, our computational results are similar to theirs. In our work, the actual spin is determined by comparing optimized relative energies under different spin at the DFT level. We scanned the energy with spin multiplicity with PBE, a pure functional which seems to perform well in the calculations of high-spin late 3d complexes.


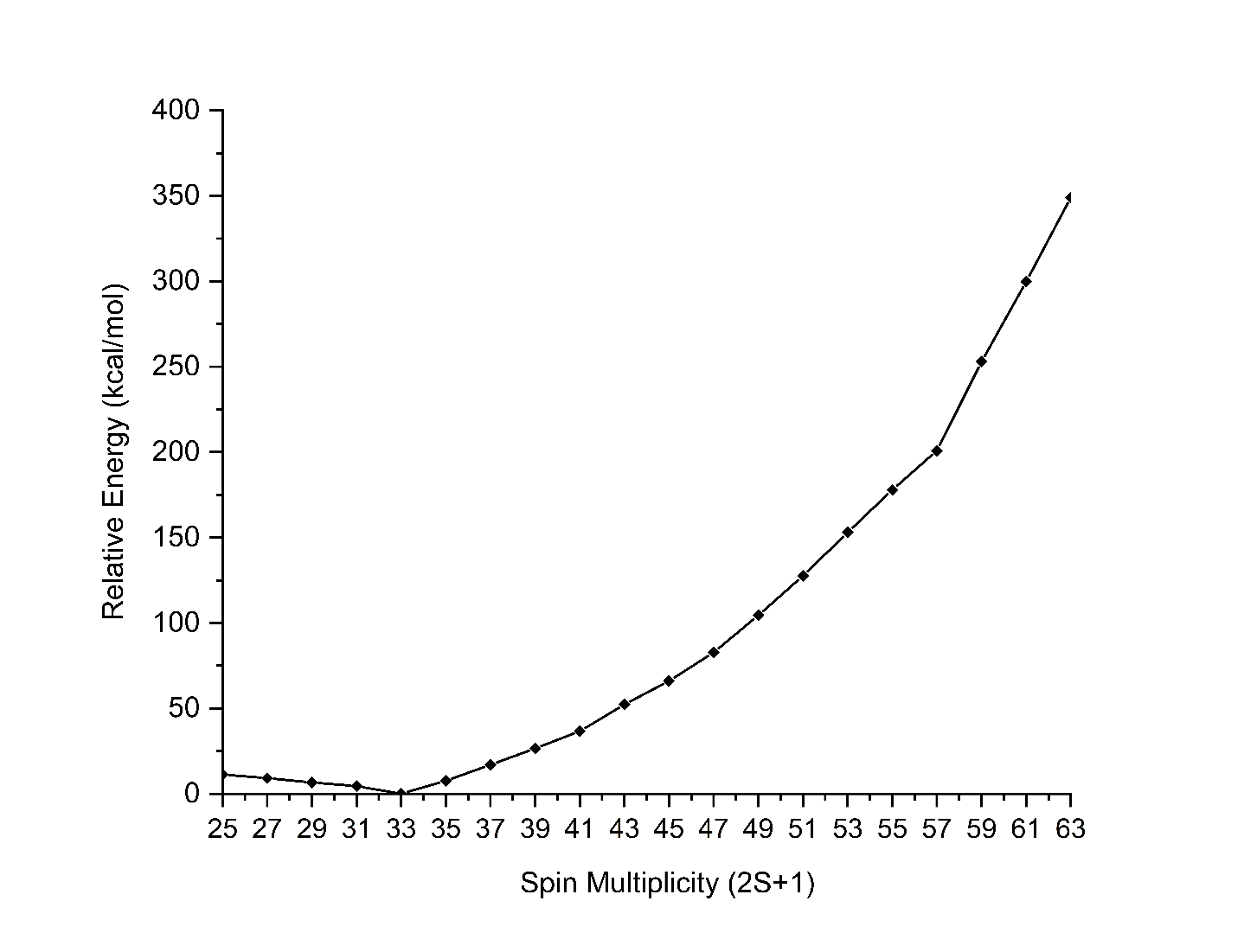


**Fig. S10.** Energy-spin curve optimized at PBE/DZP (H, B, C, N, S) +TZP (Fe, W) level.

**Note on Assignment of Oxidation State**

The (Tp*)_4_W_4_S_12_(Fe@Fe_12_) cluster as a whole is neutral, and it consists of four anionic [(Tp*)WS_3_]^4−^ ligands (Tp* = tris(3,5-dimethyl-1-pyrazolyl)borate(1−), low-valent W^3+^, and S^2-^). As the total charge state of (Tp*)_4_W_4_S_12_(Fe@Fe_12_) is (−4) × 4 + 16 = 0, the [Fe@Fe_12_] unit should have a formal +16 charge state.

Besides, the formation of [Fe@Fe_12_]^16+^ core via forming direct Fe-Fe bonds can also interpret the oxidation state of Fe. The [(Tp*)_4_W_4_S_12_(Fe@Fe_12_)] cluster can be considered as being composed of four [(Tp*)W^III^S_3_(Fe^II^_3_)]^2+^ precursor fragments and a central Fe^2+^ ion to form the [(Tp*)_4_W^III^_4_S_12_(Fe^II^@Fe^II^_12_)]^10+^ cluster with an icosahedral (Fe@Fe_12_)^26+^ cluster core. Upon formation of this tridecanuclear iron cluster, the Fe 4s-orbitals will form bonding orbitals of *a*_g_(S)+*t*_1u_(P) GOs, while the 3d-orbitals of the central Fe_i_ will be stabilized via orbital interaction with Fe 4s-based *h*_g_ GOs. The stabilization of these orbitals will cause the cluster being reduced by 10 electrons via the formation of direct Fe-Fe bonds, which leads to the (Fe@Fe_12_)^16+^ cluster core with low OS = +16/13 ( ≈ +1.23 on average). In detail, the interstitial Fe is in OS = +1, which renders the Fe_12_^15+^ charge state.

## 4 DFT exchange and correlation functionals

Various exchange and correlation functionals (BP86, PBE, BLYP, TPSS, B3LYP, PBE0, M06) were evaluated to which functional can better reproduce the crystallographic geometry. Herein, we investigated two of important geometric parameters: Fe_p_-W and Fe_i_-Fe_p_ bond lengths. The results were shown in **Fig. S11**. We concluded that BP86, PBE, TPSS functionals reproduced both bond lengths well, BLYP and M06 functionals give moderate results, while PBE0, B3LYP functionals yield unacceptable long Fe_p_-W and Fe_i_-Fe_p_ distances. Pure functionals behave better than hybrid ones on reproducing the Fe_p_-W and Fe_i_-Fe_p_ geometries of **2**. Thus, for both models, we chose structure optimized by PBE functional for further studies on electronic structures and chemical bonding.


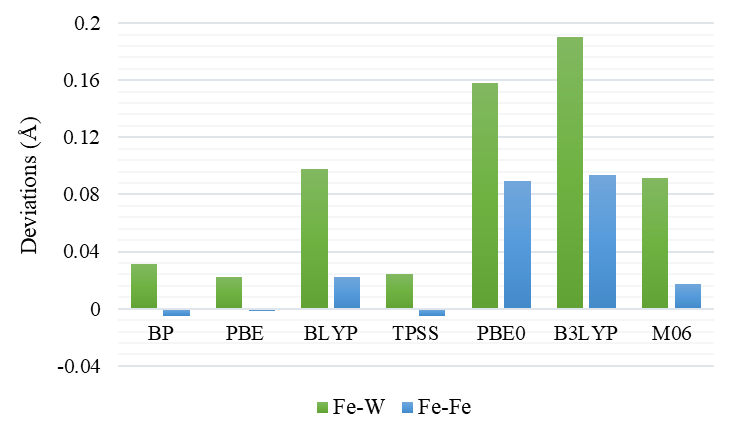


**Fig. S11.** The deviations from the crystal structure of Fe_p_-W (Fe-W) and Fe_i_-Fe_p_ (Fe-Fe) bond lengths obtained through different exchange and correlation functionals.

## 4 Geometry parameters and bond orders of 2 and 2-H

**Table S6.** Average bond length (Å) and Mayer bond order of **2** and **2-H**.

|  | Crystal Structure (Exp.) |  | PBE-optimized geometry (DFT) | | | | |
| --- | --- | --- | --- | --- | --- | --- | --- |
|  | **2** |  | **2** |  | **2-H** | | |
|  | bond length |  | bond length |  | bond length | Mayer bond order | |
|  |  |  |  |  |  | PBE | B3LYP |
| Fe_i_-Fe_p_ | 2.50 |  | 2.50 |  | 2.48 | 0.17 | 0.22 |
| Fe_i_-Fe_p_ | 2.69 |  | 2.63 |  | 2.62 | 0.42 | 0.32 |
| Fe_p_-S | 2.35 |  | 2.42 |  | 2.38 | 0.55 | 0.53 |
| Fe_p_-W | 2.70 |  | 2.72 |  | 2.63 | 0.66 | 0.54 |

## 5 Radial distribution functions (RDFs).


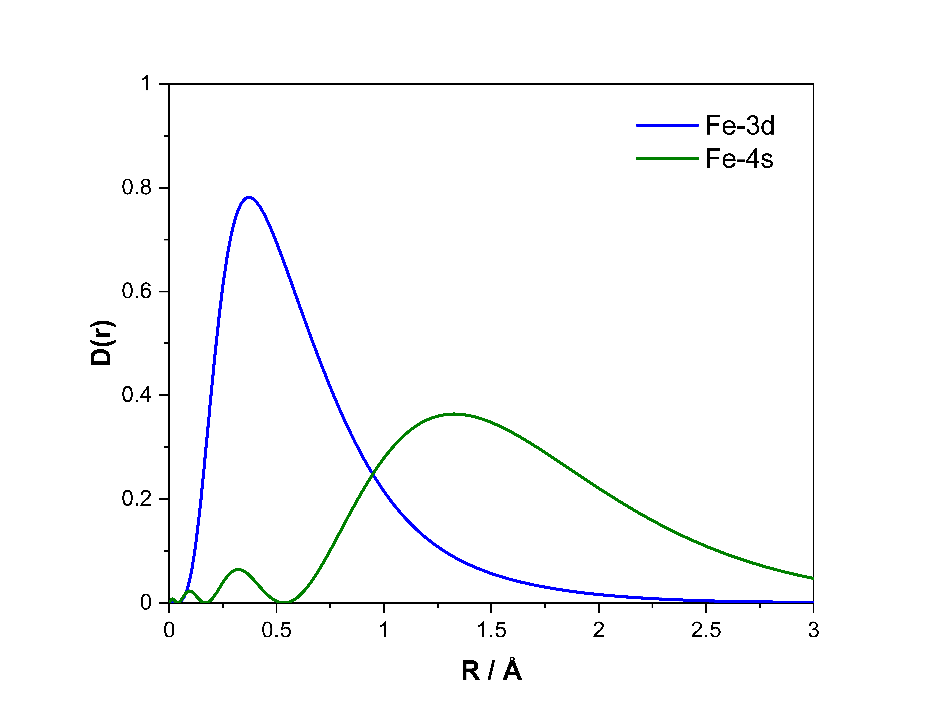


**Fig. S12.** Radial distribution functions (RDFs) of Fe-4s and Fe-3d atomic orbitals.

## 6 Atomic charge and spin population

|  |  | Atomic charge | | | Spin population | | | Natural Electron Configuration |
| --- | --- | --- | --- | --- | --- | --- | --- | --- |
|  |  | Mulliken | Bader | NPA | Mulliken | Bader | NPA |  |
| **2-H** | Fe_i_ | 1.71 | 0.11 | 0.03 | 3.08 | 2.86 | 2.99 | 3d^7.04^4s^0.85^4p^0.03^ |
|  | Fe_p_ | -0.05 | 0.40 | 0.28 | 2.52 | 2.53 | 2.43 | 3d^7.14^4s^0.49^4p^0.04^ |
|  | W | 0.60 | 1.37 | 0.29 | -0.59 | -0.50 | -0.41 | 5d^5.07^6s^0.45^6p^0.03^ |
|  | S | -0.14 | -0.71 | -0.31 | 0.08 | 0.08 | 0.13 | 3s^1.76^4p^4.50^ |
| [Fe_13_]^16+^ | Fe_i_ | 0.76 | -0.57 | -2.43 | 2.06 | 2.49 | 2.27 | 3d^7.82^4s^1.46^4p^1.10^ |
|  | Fe_p_ | 1.27 | 1.38 | 1.54 | 4.00 | 3.96 | 3.98 | 3d^5.96^4s^0.22^4p^0.06^ |
| [TpWS_3_]^4-^ | W | 0.90 | 1.64 | 0.54 | -1.28 | -0.17 | -1.12 | 5d^4.89^6s^0.45^6p^0.01^ |
|  | S | -0.99 | -1.18 | -0.92 | -0.16 | -1.17 | -0.20 | 3s^1.85^4p^5.05^ |

**Table S7.** PBE-calculated atomic charge and spin population.

## 7 Frontier Kohn-Sham canonical molecular orbitals


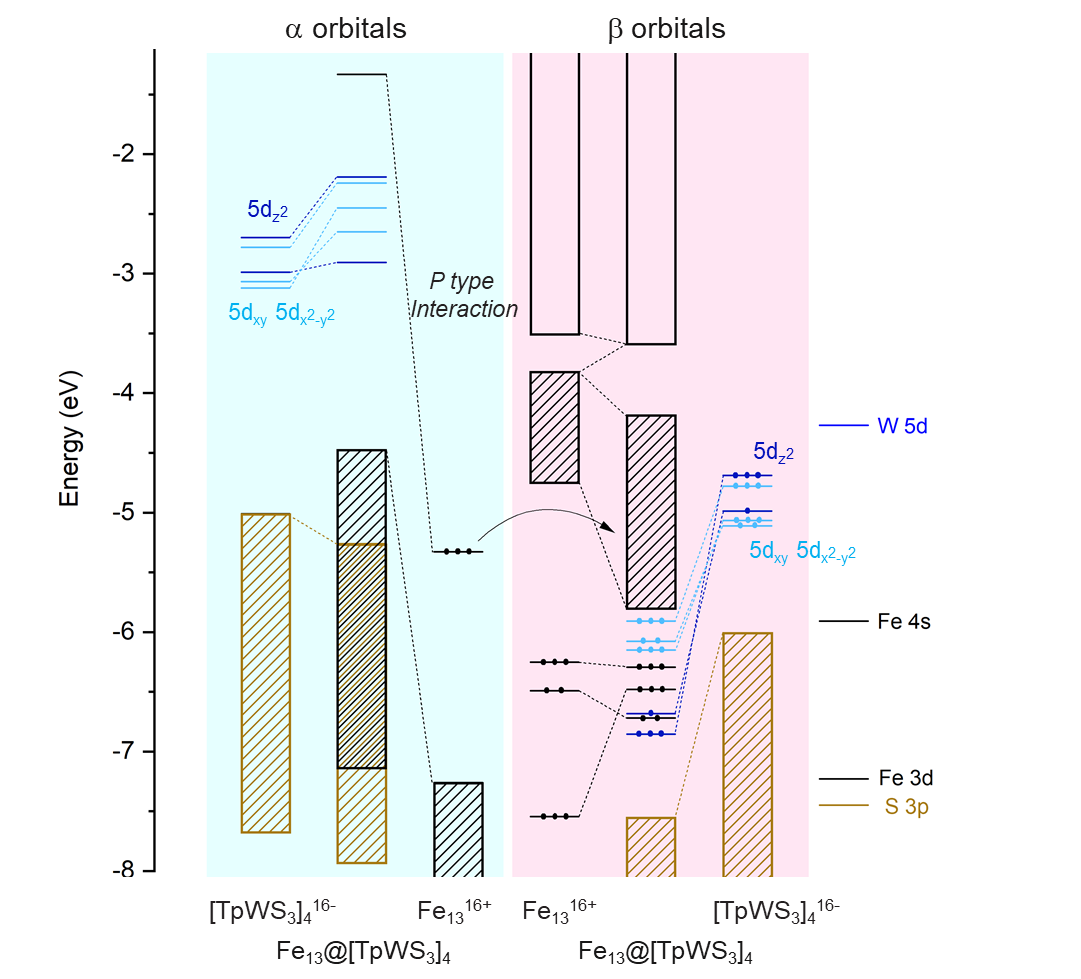


**Fig. S13.** Kohn-Sham canonical molecular orbital diagram of **2-H**.

## 8 Density of states (DOS)


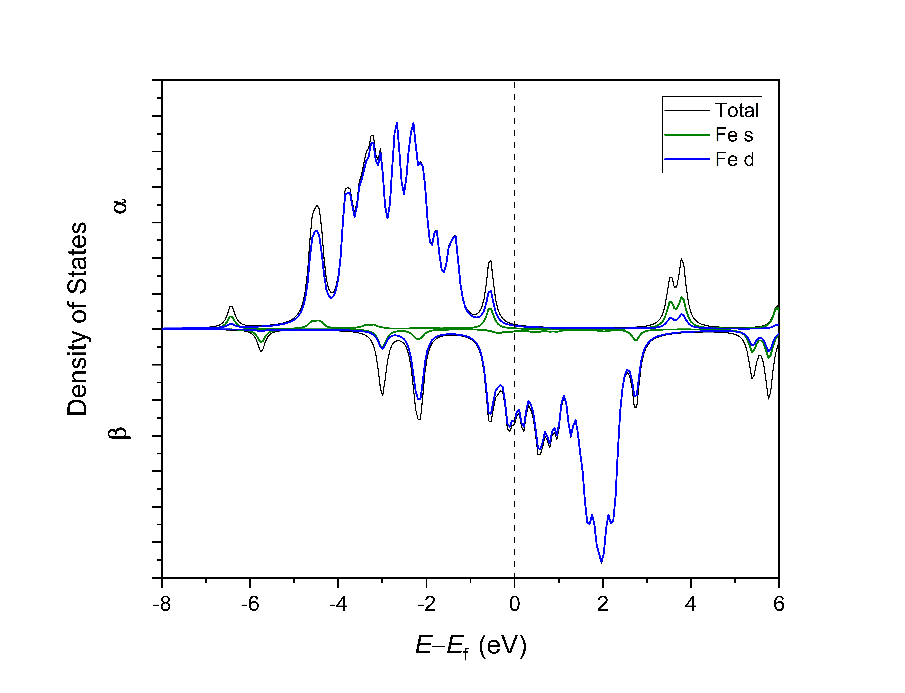


**Fig. S14.** DOS of [Fe_13_]^16+^


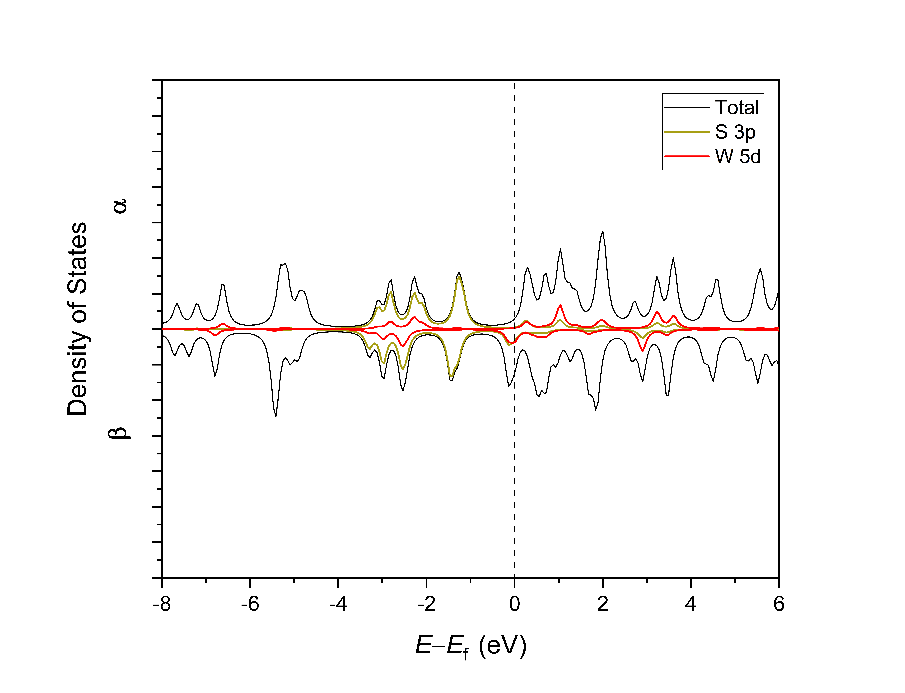


**Fig. S15.** DOS of [TpWS_3_]^4-^

## 9 PISO analysis

**Table S8.** Detailed information about the 60 major PISO pairs (contribution > 0.5%) that have had been identified, with the contribution of about 88% in the total interaction.

| Interaction type | | Number of PISO pairs | | Average Fe Contribution of PIMO (%) | | Average W Contribution of PIMO (%) | | Average S Contribution of PIMO (%) | | Contribution (%) | | |
| --- | --- | --- | --- | --- | --- | --- | --- | --- | --- | --- | --- | --- |
|  |  | α | β | α | β | α | β | α | β | α | β | total |
| Fe-W | | 12 | 12 | 75 | 52 | 12 | 22 | 11 | 22 | 19.23 | 26.92 | 46.15 |
| Fe-S | S-type | 1 | 1 | 32 | 43 | 9 | 7 | 54 | 50 | 1.19 | 2.24 | 3.44 |
|  | P-type | 3 | 3 | 39 | 37 | 5 | 4 | 54 | 57 | 6.22 | 5.86 | 12.08 |
|  | D-type | 5 | 5 | 28 | 27 | 8 | 11 | 60 | 57 | 5.9 | 5.92 | 11.83 |
|  | F-type | 3 | 3 | 21 | 20 | 13 | 15 | 59 | 58 | 1.89 | 1.64 | 3.53 |
|  | Others^a^ | 0 | 12 | - | 22 | - | 14 | - | 60 | - | 11.33 | 11.33 |
|  | Total Fe-S | 12 | 24 | 30 | 26 | 9 | 12 | 58 | 58 | 15.21 | 26.99 | 42.2 |
| Total major PISOs | | 24 | 36 | 52 | 35 | 10 | 15 | 35 | 46 | 34.45 | 53.91 | 88.36 |
| Total | | - | - | - | - | - | - | - | - | 39.16 | 60.84 | 100 |

^a^ These β interactions corresponding to S→empty Fe 3d band donation, in accord with the shift of β 3d band after ligation in DOS.


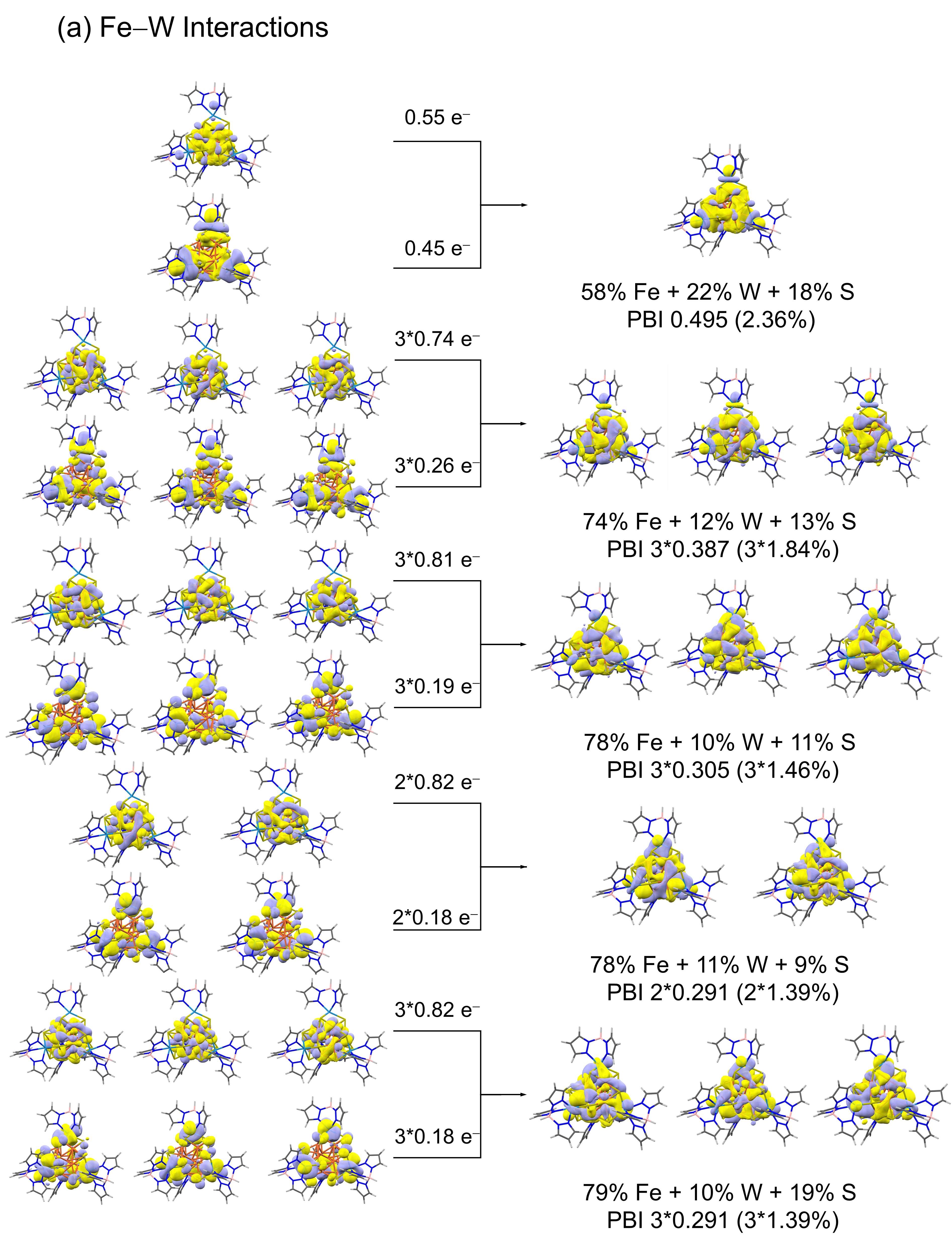


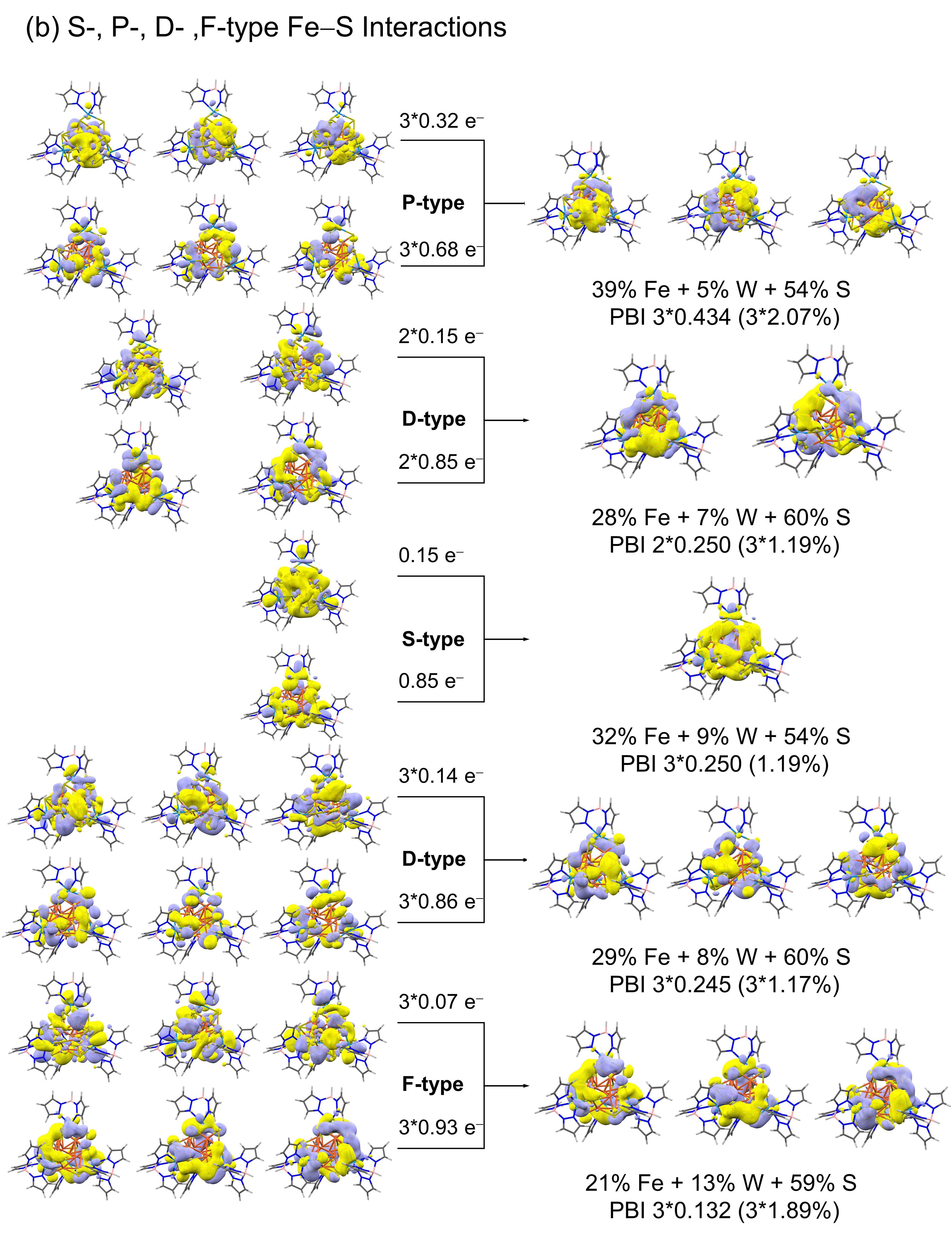


**Fig. S16.** Contours of PISO and bonding PIMO for (a) Fe-W and (b) Fe-S interactions with spin α.


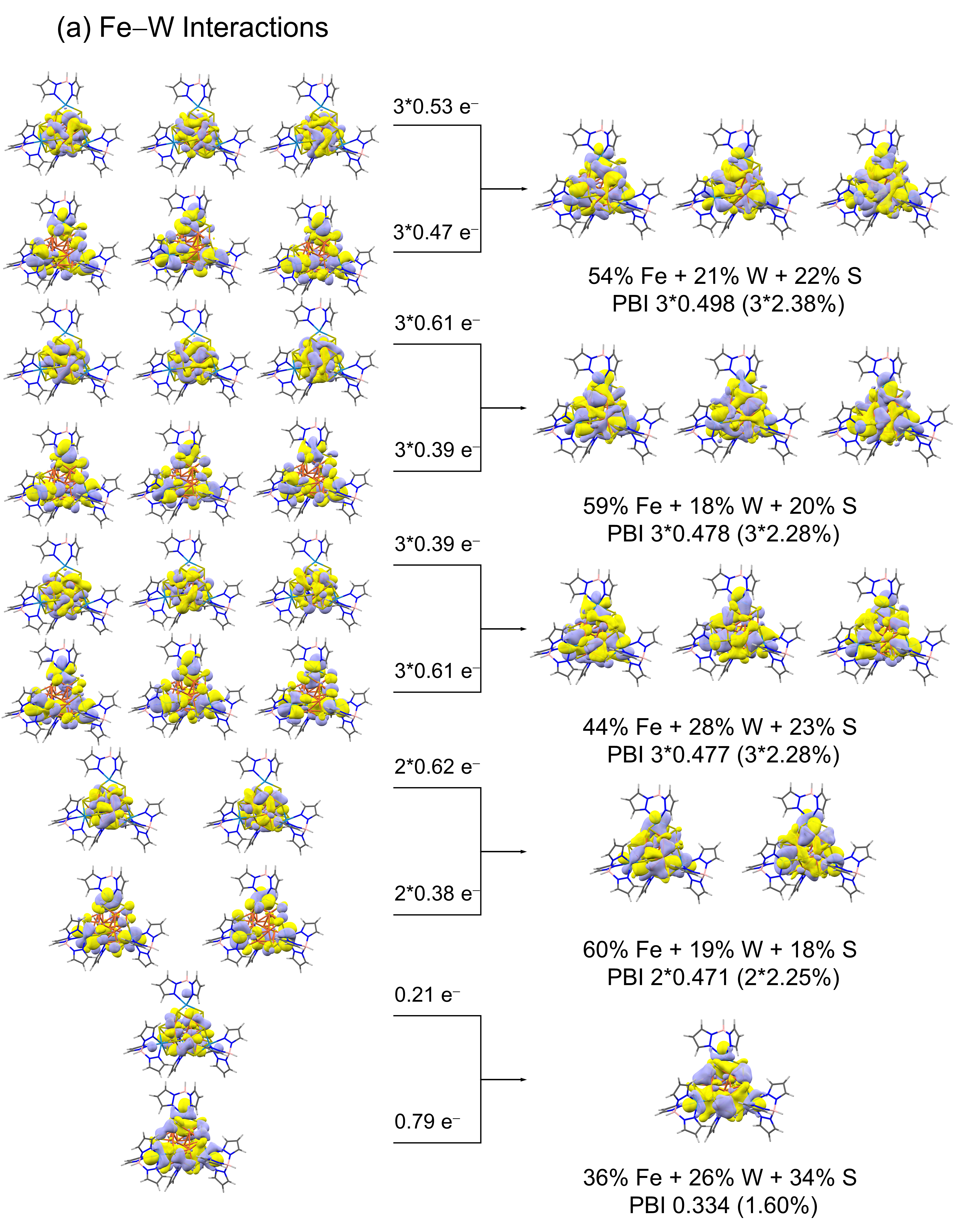


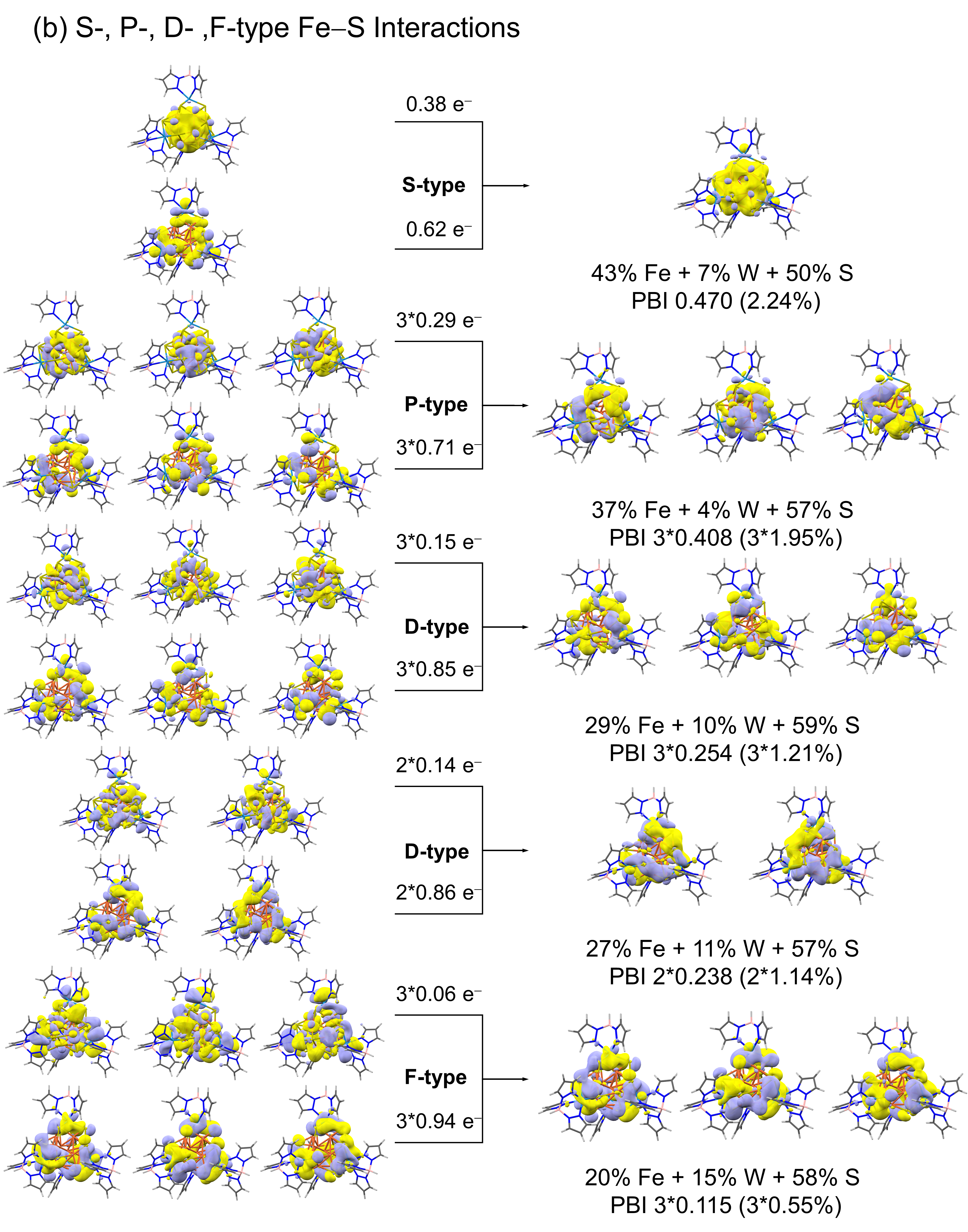


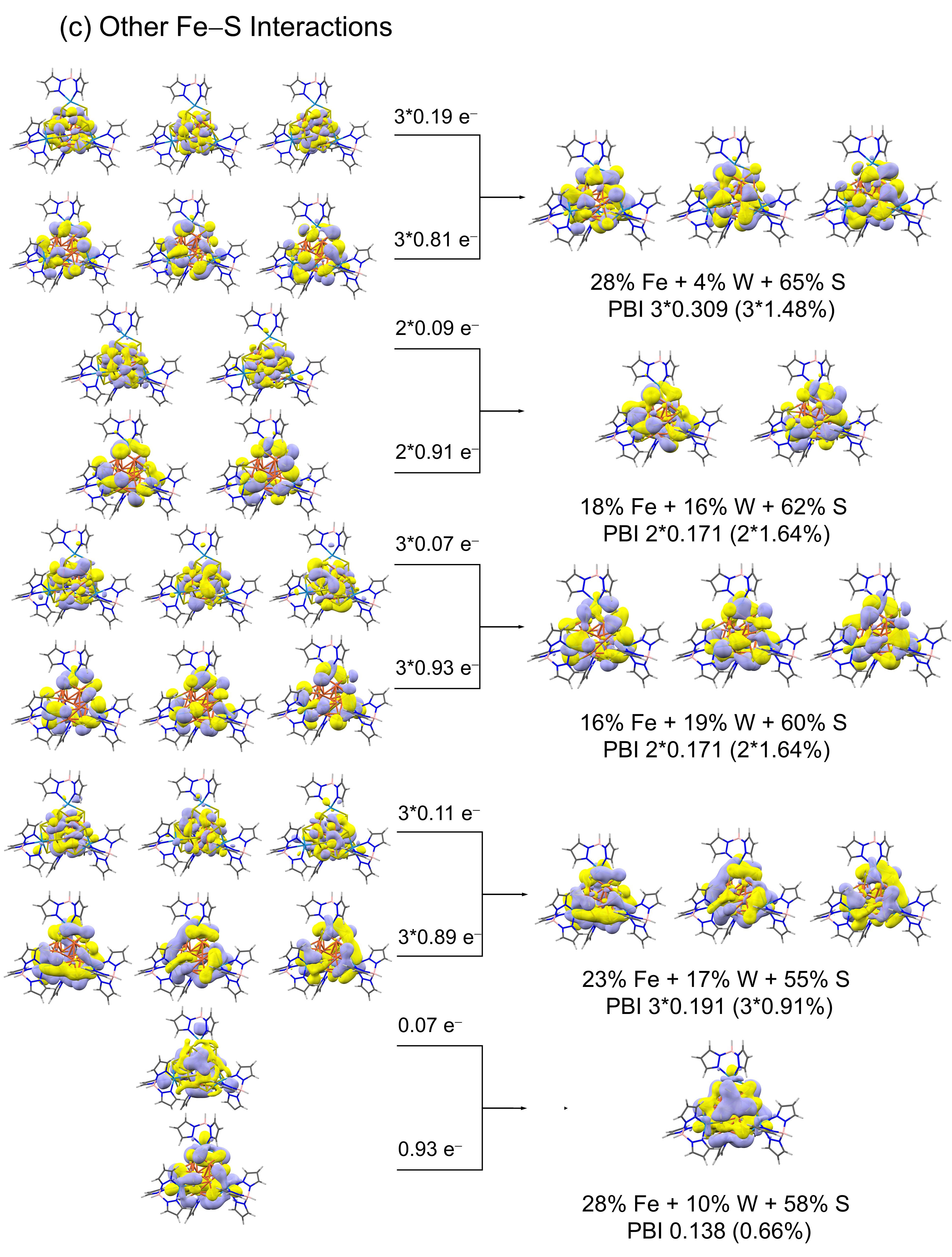


**Fig. S17.** Contours of PISO and bonding PIMO for (a) Fe-W, (b, c) Fe-S interactions with spin β.

## 10 Pipek-Mezey LMOs calculated using B3LYP functionals


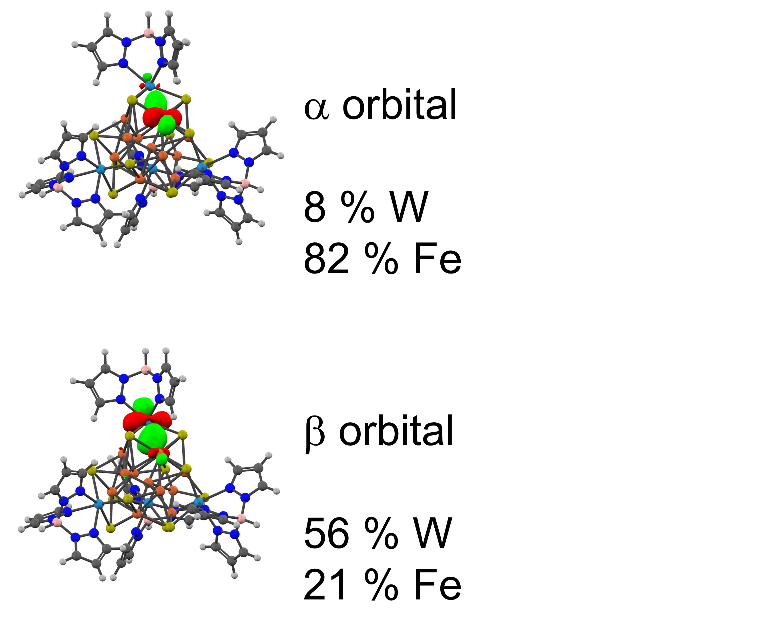


**Fig. S18.** Contours of Fe-W σ bond in Pipek-Mezey LMOs using B3LYP functional.

## 11 Spin density of 2-H


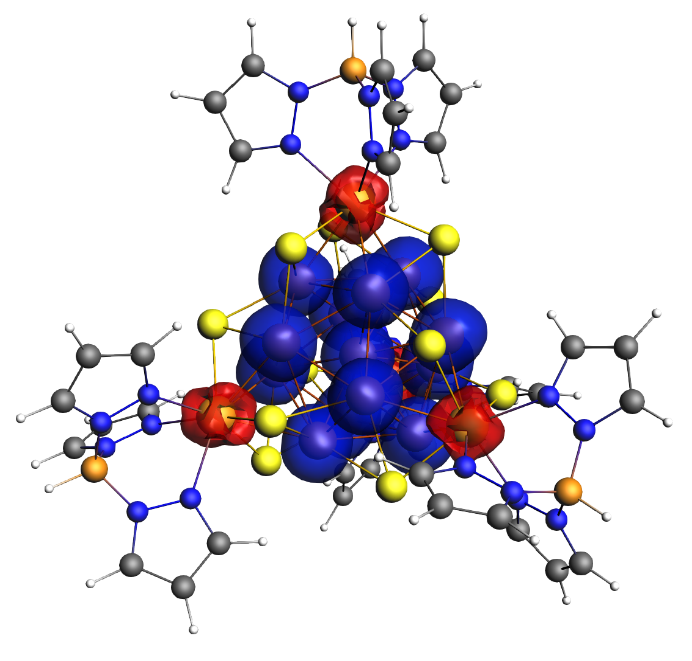


**Fig. S19.** Spin density population of **2-H**. Blue and red areas represent α and β density, respectively.

## 12 SNO analysis


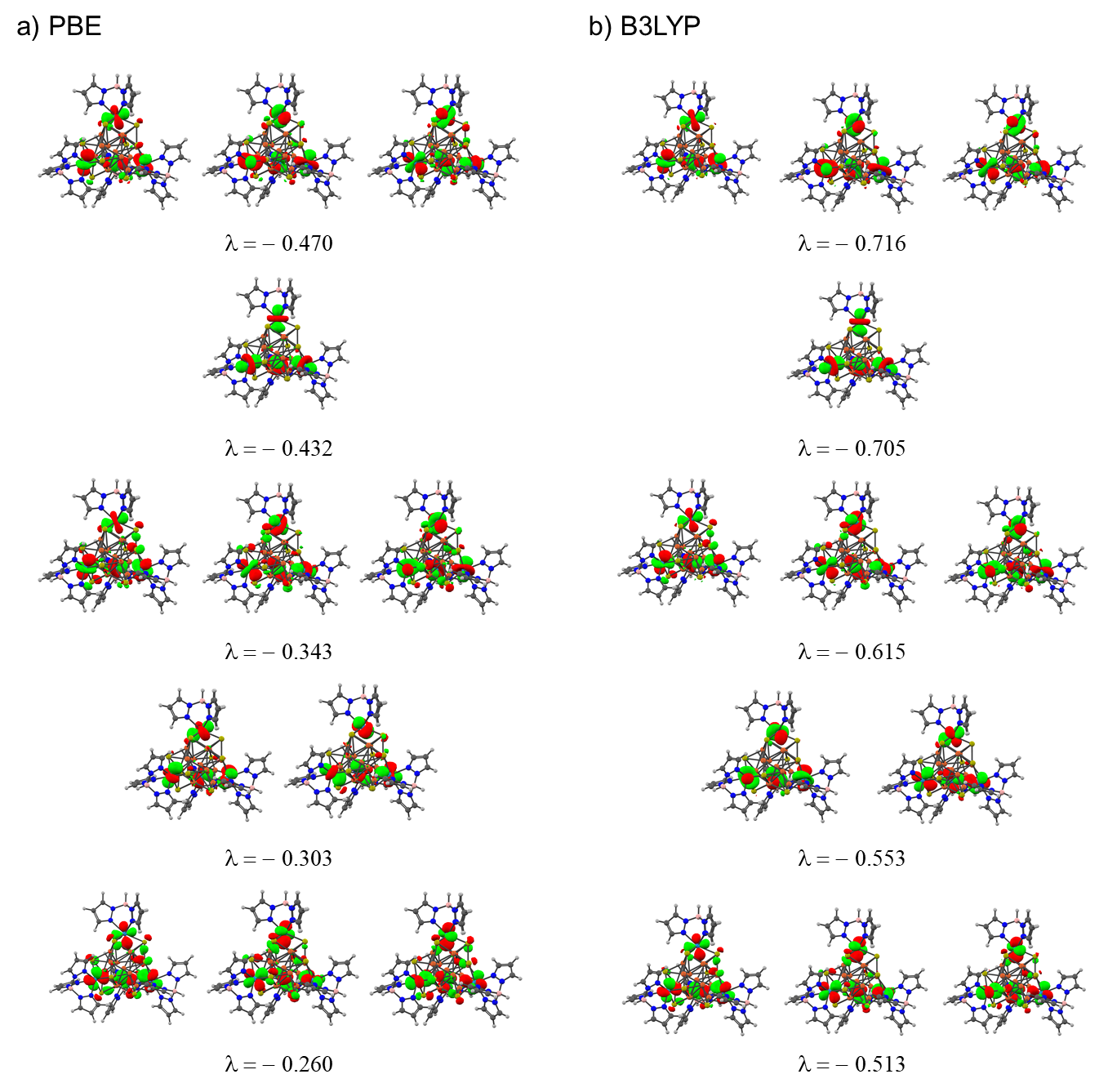


**Fig. S20.** Contours of twelve dominant SNOs with negative eigenvalues that denote W 5d orbitals.
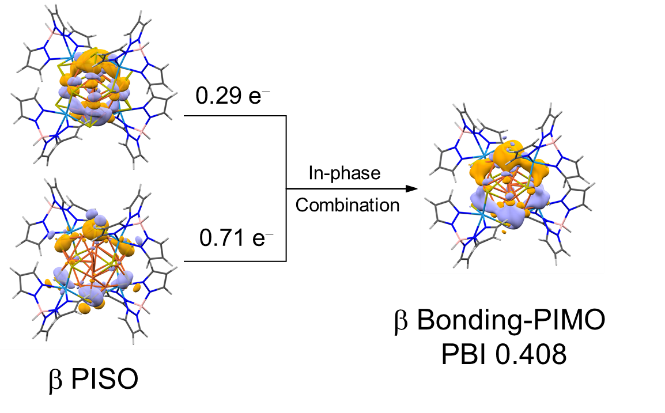


Reference

1. Kern RJ; Tetrahydrofuran complexes of transition metal chlorides. *J. Inorg. Nucl. Chem.* 1962; **24**:1105-1109.

2. Xu G, Wang Z, Ling R, et al.; Ligand metathesis as rational strategy for the synthesis of cubane-type heteroleptic iron–sulfur clusters relevant to the FeMo cofactor. *Proc. Natl. Acad. Sci. U.S.A.* 2018; **115**:5089-5092.

3. Hernández Sánchez R, Bartholomew AK, Powers TM, et al.; Maximizing Electron Exchange in a [Fe3] Cluster. *J. Am. Chem. Soc.* 2016; **138**:2235-2243.

4. Powers TM, Fout AR, Zheng S-L, et al.; Oxidative Group Transfer to a Triiron Complex to Form a Nucleophilic μ3-Nitride, [Fe_3_(μ_3_-N)]^−^. *J. Am. Chem. Soc.* 2011; **133**:3336-3338.

5. Zhao Q, Harris TD, Betley TA; [^(H)^])_2_Fe_6_(NCMe)_m_]^n+^ (m = 0, 2, 4, 6; n = -1, 0, 1, 2, 3, 4, 6): an electron-transfer series featuring octahedral Fe6 clusters supported by a hexaamide ligand platform. *J. Am. Chem. Soc.* 2011; **133**:8293-306.

6. Sánchez RH, Betley TA; Thermally Persistent High-Spin Ground States in Octahedral Iron Clusters. *J. Am. Chem. Soc.* 2018; **140**:16792-16806.

7. Muñoz SB, Daifuku SL, Brennessel WW, et al.; Isolation, Characterization, and Reactivity of Fe_8_Me_12_ ^–^: Kochi’s S =½2 Species in Iron-Catalyzed Cross-Couplings with MeMgBr and Ferric Salts. *J. Am. Chem. Soc.* 2016; **138**:7492-7495.

8. Sears JD, Muñoz Iii SB, Daifuku SL, et al.; The Effect of β-Hydrogen Atoms on Iron Speciation in Cross-Couplings with Simple Iron Salts and Alkyl Grignard Reagents. *Angew. Chem. Int. Ed.* 2019; **58**:2769-2773.

9. Lavallo V, Grubbs RH; Carbenes As Catalysts for Transformations of Organometallic Iron Complexes. *Science* 2009; **326**:559-562.

10. Toniolo D, Scopelliti R, Zivkovic I, et al.; Assembly of High-Spin [Fe_3_] Clusters by Ligand-Based Multielectron Reduction. *J. Am. Chem. Soc.* 2020; **142**:7301-7305.

11. Sheldrick G; SADABS—Bruker AXS Area Detector Scaling and Absorption. 2008.

12. Sheldrick G; SHELXT—- Integrated space-group and crystal-structure determination. [*Acta Cryst.*](https://journals.iucr.org/a) [*A*](https://journals.iucr.org/a/contents/backissues.html) 2015; **71**:3-8.

13. Sheldrick G; Crystal structure refinement with SHELXL. [*Acta Cryst.*](https://journals.iucr.org/a) *C* 2015; **71**:3-8.

14. Dolomanov OV, Bourhis LJ, Gildea RJ, et al.; OLEX2: a complete structure solution, refinement and analysis program. *J. Appl. Cryst.* 2009; **42**:339-341.

15. te Velde G, Bickelhaupt FM, Baerends EJ, et al.; Chemistry with ADF. *J. Comput. Chem.* 2001; **22**:931-967.

16. Perdew JP, Burke K, Ernzerhof M; Generalized Gradient Approximation Made Simple. *Phys. Rev. Lett.* 1996; **77**:3865-3868.

17. Van Lenthe E, Baerends EJ; Optimized Slater-type basis sets for the elements 1–118. *J. Comput. Chem.* 2003; **24**:1142-1156.

18. van Lenthe E, Baerends EJ, Snijders JG; Relativistic total energy using regular approximations. *J. Chem. Phys.* 1994; **101**:9783-9792.

19. Gaussian 16, Revision A.03, M. J. Frisch, G. W. Trucks, H. B. Schlegel, G. E. Scuseria, M. A. Robb, J. R. Cheeseman, G. Scalmani, V. Barone, G. A. Petersson, H. Nakatsuji, X. Li, M. Caricato, A. V. Marenich, J. Bloino, B. G. Janesko, R. Gomperts, B. Mennucci, H. P. Hratchian, J. V. Ortiz, A. F. Izmaylov, J. L. Sonnenberg, D. Williams-Young, F. Ding, F. Lipparini, F. Egidi, J. Goings, B. Peng, A. Petrone, T. Henderson, D. Ranasinghe, V. G. Zakrzewski, J. Gao, N. Rega, G. Zheng, W. Liang, M. Hada, M. Ehara, K. Toyota, R. Fukuda, J. Hasegawa, M. Ishida, T. Nakajima, Y. Honda, O. Kitao, H. Nakai, T. Vreven, K. Throssell, J. A. Montgomery, Jr., J. E. Peralta, F. Ogliaro, M. J. Bearpark, J. J. Heyd, E. N. Brothers, K. N. Kudin, V. N. Staroverov, T. A. Keith, R. Kobayashi, J. Normand, K. Raghavachari, A. P. Rendell, J. C. Burant, S. S. Iyengar, J. Tomasi, M. Cossi, J. M. Millam, M. Klene, C. Adamo, R. Cammi, J. W. Ochterski, R. L. Martin, K. Morokuma, O. Farkas, J. B. Foresman, and D. J. Fox, Gaussian, Inc., Wallingford CT, 2016.

20. Becke AD; Density-functional exchange-energy approximation with correct asymptotic behavior. *Phys. Rev. A* 1988; **38**:3098-3100.

21. Perdew JP; Density-functional approximation for the correlation energy of the inhomogeneous electron gas. *Phys. Rev. B* 1986; **33**:8822-8824.

22. Lee C, Yang W, Parr RG; Development of the Colle-Salvetti correlation-energy formula into a functional of the electron density. *Phys. Rev. B* 1988; **37**:785-789.

23. Tao J, Perdew JP, Staroverov VN, et al.; Climbing the Density Functional Ladder: Nonempirical Met—-Generalized Gradient Approximation Designed for Molecules and Solids. *Phys. Rev. Lett.* 2003; **91**:146401.

24. Perdew JP, Ruzsinszky A, Csonka GI, et al.; Workhorse Semilocal Density Functional for Condensed Matter Physics and Quantum Chemistry. *Phys. Rev. Lett.* 2009; **103**:026403.

25. Perdew JP, Ruzsinszky A, Csonka GI, et al.; Erratum: Workhorse Semilocal Density Functional for Condensed Matter Physics and Quantum Chemistry [*Phys. Rev. Lett*. 103, 026403 (2009)]. *Phys. Rev. Lett.* 2011; **106**:179902.

26. Becke AD; Density‐functional thermochemistry. III. The role of exact exchange. *J. Chem. Phys.* 1993; **98**:5648-5652.

27. Adamo C, Barone V; Toward reliable density functional methods without adjustable parameters: The PBE0 model. *J. Chem. Phys.* 1999; **110**:6158-6170.

28. Zhao Y, Truhlar DG; The M06 suite of density functionals for main group thermochemistry, thermochemical kinetics, noncovalent interactions, excited states, and transition elements: two new functionals and systematic testing of four M06-class functionals and 12 other functionals. *Theor. Chem. Acc.* 2008; **120**:215-241.

29. Hay PJ, Wadt WR; Ab initio effective core potentials for molecular calculations. Potentials for K to Au including the outermost core orbitals. *J. Chem. Phys.* 1985; **82**:299-310.

30. Lu T, Chen F; Multiwfn: A multifunctional wavefunction analyzer. *J. Comput. Chem.* 2012; **33**:580-592.

31. Glendening ED, Landis CR, Weinhold F; NBO 7.0: New vistas in localized and delocalized chemical bonding theory. *J. Comput. Chem.* 2019; **40**:2234-2241.
